# A Negative Feedback Loop Compromises NMD‐Mediated Virus Restriction by the Autophagy Pathway in Plants

**DOI:** 10.1002/advs.202400978

**Published:** 2024-06-21

**Authors:** Yalin Chen, Mingxuan Jia, Linhao Ge, Zhaolei Li, Hao He, Xueping Zhou, Fangfang Li

**Affiliations:** ^1^ State Key Laboratory for Biology of Plant Diseases and Insect Pests Institute of Plant Protection Chinese Academy of Agricultural Sciences Beijing 100193 China; ^2^ State Key Laboratory of Rice Biology Institute of Biotechnology Zhejiang University Hangzhou Zhejiang 310058 China

**Keywords:** autophagic degradation, nonsense mediated RNA decay, SMG7, UPF3, virus restriction

## Abstract

Nonsense‐mediated decay (NMD) and autophagy play pivotal roles in restricting virus infection in plants. However, the interconnection between these two pathways in viral infections has not been explored. Here, it is shown that overexpression of NbSMG7 and NbUPF3 attenuates cucumber green mottle mosaic virus (CGMMV) infection by recognizing the viral internal termination codon and vice versa. NbSMG7 is subjected to autophagic degradation, which is executed by its interaction with one of the autophagy‐related proteins, NbATG8i. Mutation of the ATG8 interacting motif (AIM) in NbSMG7 (SMG7mAIM1) abolishes the interaction and comprises its autophagic degradation. Silencing of NbSMG7 and NbATG8i, or NbUPF3 and NbATG8i, compared to silencing each gene individually, leads to more virus accumulations, but overexpression of NbSMG7 and NbATG8i fails to achieve more potent virus inhibition. When CGMMV is co‐inoculated with NbSMG7mAIM1 or with NbUPF3, compared to co‐inoculating with NbSMG7 in NbATG8i transgene plants, the inoculated plants exhibit milder viral phenotypes. These findings reveal that NMD‐mediated virus inhibition is impaired by the autophagic degradation of SMG7 in a negative feedback loop, and a novel regulatory interplay between NMD and autophagy is uncovered, providing insights that are valuable in optimizing strategies to harness NMD and autophagy for combating viral infections.

## Graphical abstract

Nonsense‐mediated RNA decay (NMD) factors SMG7 and UPF3 recognize viral specific RNA features to mediate viral RNA degradation for restricting virus infection. However, overexpression of SMG7 can induce autophagy, and ATG8i‐mediated the autophagic degradation of SMG7 compromises the combined anti‐viral effects of NMD and autophagy. This finding reveals that autophagy negatively regulates NMD, which can be exploited by invading virus.

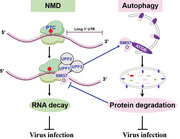

## 1 Introduction

Nonsense‐mediated decay (NMD), nonstop decay (NSD), and no‐go decay (NGD) are three preliminary known RNA quality control (RQC) systems that control the quality and stability of physiological transcripts and eliminate aberrant RNAs to avoid the accumulation of faulty or toxic proteins. NMD is triggered by ribosomes terminating at an mRNA position containing upstream open reading frames (uORFs), premature termination codons (PTCs), long 3’ untranslated regions (UTRs), or others.^[^ ^1^, ^2^, ^3^ ^]^ NSD can recognize and eliminate mRNA lacking in‐frame termination codons, while NGD degrades mRNA by specific structure features.^[^ ^4^, ^5^, ^6^ ^]^ In plants, up‐frameshift (UPF) proteins, UPF1, UPF2, and UPF3, and the suppressor with morphogenetic effect on genitalia 7 (SMG7) constitute the NMD core machinery.^[^ ^7^, ^8^ ^]^ The UPF proteins interact to form a complex to recruit an SMG7‐induced exonucleolytic pathway, including 5’‐3’ and 3’‐5’ cytoplasmic RNA decay pathways, to degrade NMD targets.^[^ ^6^, ^9^, ^10^ ^]^ To maximize encoding capability, RNA viruses often include internal termination codons (iTCs), making them ideal targets for NMD. For example, several positive single‐stranded RNA viruses (+ssRNA), including potato virus X (PVX), turnip crinkle virus, semliki forest virus, murine hepatitis virus, and Zika virus, have been identified to be susceptible to NMD.^[^ ^11^, ^12^, ^13^, ^14^ ^]^ Using PVX‐GFP as a model, mutation of a major NMD effector, *UFP1*, in *Arabidopsis thaliana* enhanced PVX‐GFP accumulation and transient overexpression of wild‐type rather than dominant‐negative UPF1 restricted virus infection in *Nicotiana benthamiana*.^[^ ^11^ ^]^ A recent report showed that the Pelota‐Hbs1 RNA surveillance complex‐mediated NGD and NSD attenuated the disease of several viruses in the family *Potyviridae* by recognizing the viral conserved GA motif and mediating viral RNA degradation.^[^ ^15^ ^]^ Furthermore, NMD factors, including UPF3 and SMG7, recognized the m^6^A readers‐viral m^6^A RNA complex for viral RNA degradation to limit virus infection in plants.^[^ ^16^ ^]^ Thus, RQC‐dependent viral RNA degradation is thought to be a pivotal viral restriction mechanism.

Autophagy is an evolutionarily conserved intracellular mechanism in eukaryotes that mediates the degradation of cytoplasmic components and damaged organelles through lysosomes or vacuoles. This process can be induced by starvation salt, drought, oxidative stress, and pathogen invasion in plants.^[^ ^17^, ^18^, ^19^, ^20^, ^21^ ^]^ Emerging evidence has shown that autophagy could limit the infection of plant viruses, including cauliflower mosaic virus (CaMV),^[^ ^22^ ^]^ cotton leaf curl Multan virus (CLCuMuV),^[^ ^23^ ^]^ tomato leaf curl Yunnan virus (TLCYnV),^[^ ^24^ ^]^ and turnip mosaic virus (TuMV), cucumber green mottle mosaic virus (CGMMV), and pepino mosaic virus,^[^ ^25^, ^26^ ^]^ through targeting specific viral proteins for autophagic degradation.^[^ ^26^, ^27^, ^28^ ^]^ For example, Beclin1 (ATG6), a core autophagy‐related gene (ATG), could interact with the RNA‐dependent RNA polymerase (RdRP) of several RNA viruses to guide their autophagic degradation to restrict virus infection.^[^ ^25^ ^]^ However, to successfully establish infection, viruses have evolved sophisticated strategies to avoid, manipulate, or antagonize host defense pathways, including autophagy.^[^ ^26^, ^27^, ^29^ ^]^ For example, the barley stripe mosaic virus γb protein and geminiviruses encoded C2 proteins could inhibit autophagy activity for facilitating virus infection by impairing the ATG7‐ATG8 interaction.^[^ ^29^, ^30^ ^]^ In virus‐infected cells, it is considered that both NMD and autophagy could be activated to restrict virus infection. In contrast, the mechanisms underlying the crosstalk between these two pathways are unknown.

In this study, we use CGMMV, a +ssRNA virus belonging to *Tobamovirus* of *Virgaviridae*, as a model to investigate the roles and crosstalk effects of NMD and autophagy in virus infection. CGMMV, a seriously devastating plant RNA virus in many countries, causes severe symptoms in infected plants, including mosaic leaves, dwarfing, and fruit drooping, resulting in the enormous economic losses.^[^ ^31^ ^]^ The hosts of CGMMV include many crops of *Cucurbitaceae*, as well as model plants such as *Chenopodium amaranticolor, Datura stramonium*, and *Nicotiana benthamiana*.^[^ ^31^, ^32^ ^]^ The viral genome length of CGMMV is 6423 nt and encodes four known proteins, a 129 kDa RdRP, a 186 kDa RdRP, a 29 kDa Move Protein (MP), and a 17.3 kDa Coat Protein (CP).^[^ ^33^ ^]^ Among these, the 186 kDa RdRP (hereafter referred to as RdRP‐L) is an iTC read‐through product of the 129 kDa RdRP (hereafter referred to as RdRP‐S). These two RdRPs are thought to be involved in virus replication and infection.^[^ ^31^ ^]^ Here, we show that NMD factors NbSMG7 and NbUPF3 fail to interact directly with CGMMV‐encoded proteins. Still, they recognize viral RNAs with iTC as a substrate and mediate their degradation to restrict virus infection. We find that CGMMV infection triggers autophagy and decreases the accumulation of NbSMG7. NbSMG7, rather than NbUPF3, is degraded by autophagy via its ATG8 interacting motif (AIM) interacting with NbATG8i. Interestingly, silencing both *NbSMG7* and *NbATG8i* together leads to higher CGMMV RNA accumulations than silencing each individually. In comparison, overexpression of both NbSMG7 and NbATG8i fails to achieve more potent inhibition of virus infection than overexpression of either gene alone. At the same time, when the CGMMV infectious clone was co‐inoculated with NbSMG7^mAIM1^ or with NbUPF3, compared to co‐inoculating with NbSMG7 in NbATG8i overexpression transgene plants, the inoculated plants display milder virus symptoms and accumulate less virus titers. Therefore, this study reveals the role of NMD factors in CGMMV infection and highlights the crosstalk of NMD and autophagy during virus infection, providing new insight into complex plant‐pathogen interactions.

## 2 Results

### 2.1 Molecular Characterization of NbSMG7 and NbUPF3

**Figure 1:**
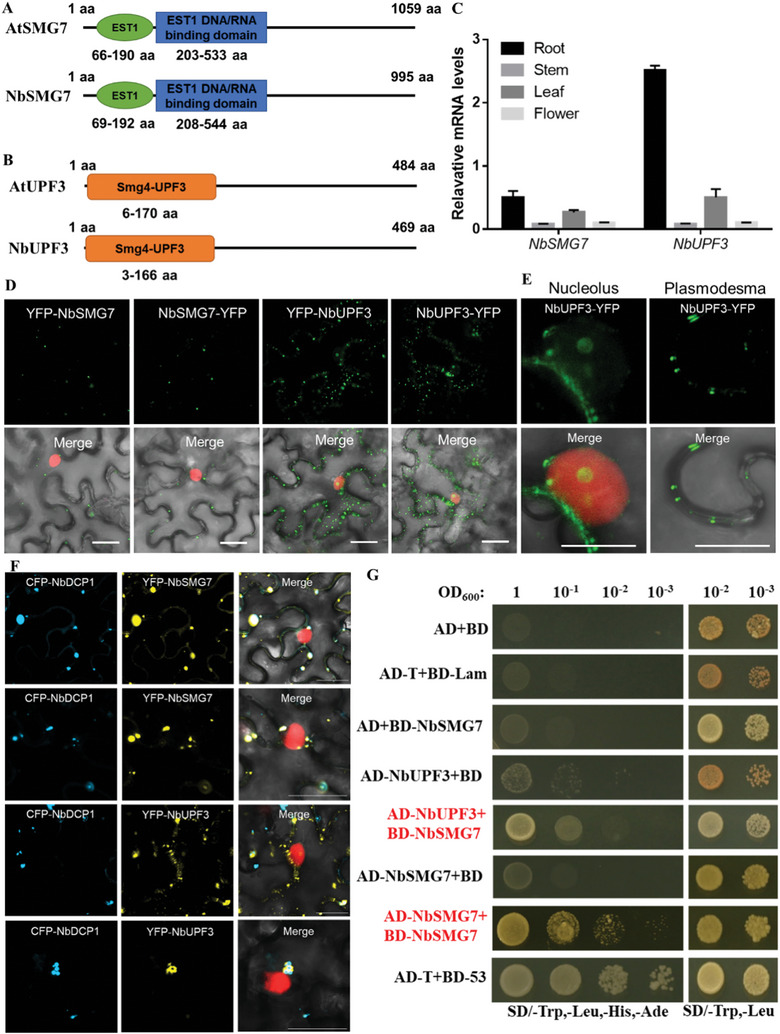
Molecular characterization and interactions of NbSMG7 and NbUPF3. A) The linear diagram represents the domain structure of NbSMG7 and AtSMG7, EST1: Telomerase activating protein, EST1‐DNA‐bind: Est1 DNA/RNA binding domain. B) The linear diagram represents the domain structure of NbUPF3 and AtUPF3, Smg4‐UPF3: Smg‐4/UPF3 family. C) RT‐qPCR analysis of *NbSMG7* and *NbUPF3* expression levels in different tissues of *N. benthamiana*. Expression was normalized against *NbActin* transcripts, which serve as an internal standard. Each mean value was derived from three independent experiments (*n* = 3 samples). Values represent the mean ± standard deviation (SD). Statistical analysis was performed using Student's *t‐*test (two‐sided, ^**^ *p* < 0.01). D) Micrographs showing cells from leaves of RFP‐H2B transgenic *N. benthamiana* expressing YFP‐NbSMG7, NbSMG7‐YFP, YFP‐NbUPF3, NbUPF3‐YFP at 32 hpi. Bars = 20 µm. E) The localization of NbUPF3‐YFP in the nucleolus and plasmodesma was observed by confocal at 32 hpi. Bars = 50 µm. F) Co‐localization of NbSMG7‐YFP, or NbUPF3‐YFP with the NbDCP1‐CFP in leaves of RFP‐H2B transgenic *N. benthamiana* by confocal microscopy. Bars = 20 µm. G) Yeast two‐hybrid assays using NbSMG7 and NbUPF3 or NbSMG7 and NbSMG7. Yeast cells co‐transformed with AD‐T7‐T+BD‐T7‐53 were a positive control, and cells co‐transformed with AD‐T7‐T+BD‐T7‐Lam, or with empty vectors, pGBKT7 (BD) and pGADT7 (AD) served as negative controls. BD, GAL4 DNA binding domain; AD, GAL4 activation domain.

NMD is an essential eukaryotic RNA surveillance pathway, which has been shown to restrict virus infection in plants, and the role of the main NMD effector UPF1 in anti‐viral immunity has been reported.^[^ ^11^, ^34^ ^]^ Mammalian and plant NMD share significant similarities, but they differ in terms of specific mechanisms and regulation. There are distinct features of plant NMD.^[^ ^8^ ^]^ For instance, several homologs of mammalian NMD factors, including SMG5, SMG6, SMG8, and SMG9, have not been identified in plants. In mammals, SMG6‐dependent endonucleolytic cleavage is a crucial step in NMD. In contrast, in plants, NMD utilizes the SMG7‐induced exonucleolytic pathway (5'‐3' RNA decay and 3'‐5' RNA decay by recruiting decapping enzymes and deadenylating enzymes) for target degradation.^[^ ^8^ ^]^ Therefore, we initiated this study by cloning other key NMD factors from *N. benthamiana* plants, NbSMG7 and NbUPF3, to investigate their potential biological functions. Sequence analysis revealed that the open reading frames (ORFs) of *NbSMG7* and *NbUPF3* contain 2982 nt (GenBank accession number: MW343792) and 1410 nt (GenBank accession number: MW380239), respectively. *NbSMG7* encodes a 993‐amino acid (aa) protein with a structure similar to *AtSMG7*. It contains a telomerase activating domain EST1, which recruits or activates telomerase at the site of polymerization, and an EST1 DNA/RNA binding domain (**Figure** **1**a). *NbUFP3* cDNA encodes a 469‐amino acid (aa) protein with a structure similar to AtUPF3 and contains a Smg4‐UPF3 domain involved in NMD (Figure 1B). The amino acid sequences of NbSMG7 and NbUPF3 share 51.5% and 50.6% identity with their counterparts, AtSMG7 and AtUPF3, from Arabidopsis.

To gain insight into the expression patterns of *NbUPF3* and *NbSMG7*, reverse transcription real‐time quantitative PCR (qRT‐qPCR) was used to analyze their mRNA levels using total RNA isolated from different *N. benthamiana* tissues. *NbUPF3* and *NbSMG7* expression patterns were very similar, with the highest expression level in the root tissue, the higher expression level in the stem tissue than in the leaf tissue, and the lowest in the flower tissue (Figure 1C).

To examine the subcellular localization of these two NMD genes, their coding regions were fused to in‐frame upstream/downstream of the coding sequence of yellow fluorescence protein (YFP) by gateway technology. The recombinant constructs were transiently expressed in transgenic *N. benthamiana* plants expressing RFP‐H2B as a nuclear marker, and fluorescence was examined in agroinfiltrated transgenic leaves at 32 h post infiltration (hpi) by confocal microscopy (Figure 1D). YFP‐NbSMG7 and NbSMG7‐YFP were present predominantly in the cytoplasm, forming small round granules. YFP‐NbUPF3 and NbUPF3‐YFP formed small round granules in the cytoplasm, the nucleus, and also at the plasmodesmata (Figure 1E). Co‐localization assays of NbSMG7‐YFP, or NbUPF3‐YFP with the mRNA processing (P)‐body marker, NbDCP1‐CFP, showed that NbSMG7‐YFP rather than NbUPF3‐YFP was obviously localized in the P‐body (Figure 1F).

SMG7 and UPFs cooperatively function in the NMD pathway.^[^ ^10^ ^]^ To determine whether NbSMG7 and NbUPF3 interact with each other directly, we performed yeast two‐hybrid (Y2H) assays. However, there was no direct interaction between NbSMG7 and NbUPF3 in yeast cells, as shown in Figure 1G.

### 2.2 Transient Expression of NbSMG7 and NbUPF3 Inhibits CGMMV RNA Accumulations by Targeting Virus iTC for RNA Degradation

It has been shown that AtUPF1 and AtUPF3 are required for NMD, and an inverted repeat (IR) transgene‐induced gene silencing is impaired in *upf1*‐*5 *mutants, indicating a connection between UPF1 and RNA interference in plants.^[^ ^35^ ^]^ To explore whether the *N. benthamiana* NMD factors NbSMG7 and NbUPF3 affect RNA silencing. *Agrobacterium* cultures expressing 35S‐GFP and Myc‐tagged NbSMG7 or NbUPF3 were co‐infiltrated into 16c *N. benthamiana* leaves. As controls, leaves were co‐infiltrated with 35S‐GFP and Myc‐tagged GUS, or the vector for expression of the p19 protein (P19) of tomato bushy stunt virus (TBSV), a well‐known RNA silencing suppressor. Figure S1A (Supporting Information) shows that no evident GFP silencing suppression was observed in NbSMG7 or NbUPF3 over‐expressed local leaf patches and systemic leaves. RT‐qPCR and immunoblotting results support the overexpression of NbSMG7 or NbUPF3 could not suppress GFP silencing in 16c plants (Figure S1B–D, Supporting Information), indicating that NbSMG7 or NbUPF3 might have no apparent role in RNA silencing.

**Figure 2:**
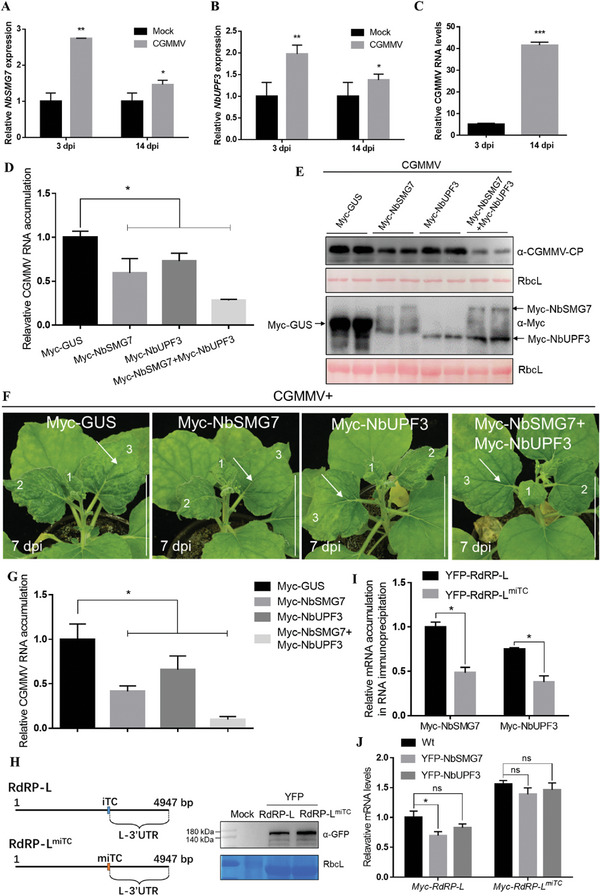
CGMMV infection upregulates the NMD pathway in *N. benthamiana*, and overexpression of NbSMG7 or/and NbUPF3 inhibits CGMMV infection. A,B) The expression levels of *NbSMG7* (A) and *NbUPF3* (B) were analyzed by RT‐qPCR in Mock (infiltration buffer) or CGMMV‐infiltrated *N. benthamiana* leaves at 3 dpi or upper new leaves at 14 dpi. *NbActin* was used as an internal standard. Each mean value was calculated based on three independent biological repeats (*n* = 3 samples). C) RT‐qPCR analyzed the CGMMV RNA levels on CGMMV‐infiltrated *N. benthamiana* leaves at 3 dpi or upper new leaves at 14 dpi. *NbActin* was used as an internal standard. Each mean value was calculated based on three independent biological repeats (*n* = 3 samples). D) RT‐qPCR analysis of CGMMV RNA levels. RNA was extracted from leaves agroinfiltrated with CGMMV and Myc‐tagged GUS, NbSMG7, NbUPF3, or NbSMG7 and NbUPF3 at 60 hpi. E) Immunoblotting of total protein extracts from the *N. benthamiana* leaves agroinfiltrated with Myc‐GUS or the plasmids indicated. Immunoblotting was performed using anti‐CGMMV CP or anti‐Myc antibodies. Each experiment was repeated at least three times. The large RuBisCO subunit (rbcL) was stained with Ponceau S to indicate equal protein loading. F) Symptoms of CGMMV‐infected plants at 14 dpi. The plants were inoculated with CGMMV and Myc‐tagged GUS, NbSMG7, NbUPF3, NbSMG7, and NbUPF3 at 14 dpi. G) RT‐qPCR analysis of CGMMV RNA accumulations in plants indicated in (F). Values represent means ± SD relative to plants infected with CGMMV and Myc‐GUS. H) Schematic diagram on the mutation of RdRP‐L^miTC^. Immunoblotting of total protein extracts from the *N. benthamiana* leaves agroinfiltrated with YFP‐RdRP‐L or YFP‐RdRP‐L^miTC^. Immunoblotting was performed using anti‐GFP antibodies. I) RNA immunoprecipitation (RIP) coupled with qRT‐PCR assays of Myc‐NbSMG7 and Myc‐NbUPF3 associating with YFP‐RdRP‐L and YFP‐RdRP‐L^miTC^ RNA in vivo at 24 hpi. J) qRT‐PCR analysis of Myc‐RdRP‐L and Myc‐RdRP‐L^miTC^ RNA transcripts in wild‐type (Wt), transgenic YFP‐NbSMG7‐L5 and YFP‐NbUPF3‐L7 transgenic *N. benthamiana* leaves at 60 hpi. The error bars indicate mean ± SD (*n* = 3); asterisks indicate a statistically significant difference according to Student's *t*‐test (^**^ *p* < 0.01; ^*^ *p* < 0.05), and ns means no significant difference (A–C, D, G, I,J).

To clarify the role of NMD in CGMMV infection, we investigated the expression levels of *NbSMG7* or *NbUPF3* by using RT‐qPCR in CGMMV‐infected local and systemic leaves of *N. benthamiana* plants at 3 and 14 dpi, respectively (**Figure** **2**A,B). The expression levels of all these two NMD genes were significantly upregulated in both local and systemic leaves in response to CGMMV infection in *N. benthamiana* plants (Figure 2A,B), which was negatively regulated by the amount of CGMMV after 3 dpi (Figure 2C). We further checked whether CGMMV‐encoded proteins interact with NbSMG7 or NbUPF3. The four proteins encoded by CGMMV (RdRP‐L, RdRP‐S, MP, and CP), NbSMG7, and NbUPF3, were fused to the GAL4 transcription activation domain (AD) and the GAL4 DNA binding domain (BD). The Y2H assays between CGMMV‐encoded four proteins and NbSMG7 or NbUPF3 were performed. As summarized in Figure S2A,B (Supporting Information), regardless of whether AD or BD fusions were used for the assay, positive interactions were only found in yeast cells co‐transformed with positive control AD‐T and BD‐53. Immunoblotting analysis confirmed the protein expression of the four CGMMV‐encoded proteins in co‐transformed yeast cells in other experiments.^[^ ^25^ ^]^ These results indicate that CGMMV infection triggers the NMD pathway, but CGMMV‐encoded proteins do not directly interact with NMD factors SMG7 or UPF3.

The CGMMV genome contained iTCs, which can be ideal targets of NMD. To examine whether NbSMG7 /and NbUPF3 affect CGMMV RNA accumulation, CGMMV was co‐infiltrated into *N. benthamiana* leaves with Myc‐tagged GUS, NbSMG7, NbUPF3, or NbSMG7 and NbUPF3. At 60 hpi, leaf patches co‐ infiltrated with either NbSMG7, NbUPF3, or both showed reduced levels of viral RNA compared to those co‐ infiltrated with Myc‐tagged GUS (Figure 2D). Immunoblotting assays showed the expression of Myc‐tagged GUS, NbSMG7, and NbUPF3 and confirmed the lower viral CP accumulations in agroinfiltrated *N. benthamiana* leaves co‐expressing with NbSMG7, NbUPF3, or NbSMG7 and NbUPF3 (Figure 2E). The above‐infiltrated plants were maintained to monitor viral symptom development and analyze viral RNA accumulation in newly emerged leaves. Consistently, overexpression of NbSMG7, NbUPF3 or NbSMG7, and NbUPF3 attenuated viral symptoms and systemic viral infection and decreased viral RNA accumulations (Figure 2F,G).

**Figure 3:**
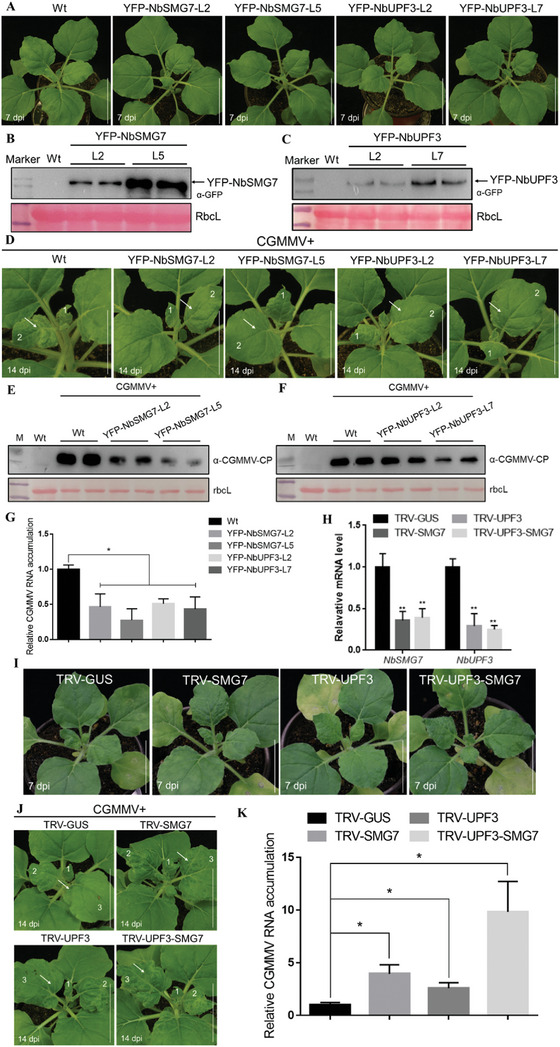
NbSMG7 or/and NbUPF3 negatively regulated by CGMMV infection in *N. benthamiana*. A) Comparison of phenotypes among T1 generation YFP‐NbSMG7 and YFP‐NbUPF3 transgenic plants and wild‐type *N. benthamiana*. B,C) Immunoblotting of total protein extracts from the YFP‐NbSMG7 and YFP‐NbUPF3 transgenic *N. benthamiana* leaves. Immunoblotting was performed using anti‐GFP antibodies. The RbcL was stained with Ponceau S to indicate equal protein loading. D) Symptoms of CGMMV‐infected plants at 14 dpi. The Wt, YFP‐NbSMG7, or YFP‐NbUPF3 transgene plants were inoculated with CGMMV at 14 dpi. E,F) Immunoblotting of total protein extracts from the YFP‐NbSMG7 (E) and YFP‐NbUPF3 (F) transgenic *N. benthamiana* leaves. Immunoblotting was performed using anti‐CGMMV CP antibodies. The RbcL was stained with Ponceau S to indicate equal protein loading. G) qRT‐PCR analysis of CGMMV RNA accumulations in plants indicated in (D). H) Confirmation of knockdown of *NbSMG7*, *NbUPF3*, or both in upper new leaves of *N. benthamiana* plants at 14 dpi (H) by qRT‐PCR. Double asterisks indicate significant differences in *NbSMG7* expression level between TRV‐NbSMG7 or TRV‐NbSMG7‐NbUPF3 and TRV‐GUS‐treated plants, or *NbUPF3* expression level in between TRV‐NbUPF3, or TRV‐NbSMG7‐NbUPF3 and TRV‐GUS‐treated plants. I) The phenotype in TRV‐GUS, TRV‐NbSMG7, TRV‐NbUPF3, or TRV‐NbSMG7‐NbUPF3 treated plants at 14 dpi. J) CGMMV symptoms in plants pre‐inoculated with TRV1 and TRV2‐GUS, TRV2‐NbSMG7, TRV2‐NbUPF3, or TRV2‐NbSMG7‐NbUPF3 for 7 days and then infected by CGMMV infectious clones. Plants were photographed at 14 dpi. K) qRT‐PCR analysis of CGMMV genomic RNA in the above plants. RNA was extracted from the systemically infected leaves at 14 dpi. The values are presented as means ± SD relative to CGMMV‐inoculated TRV‐GUS‐treated leaves at 14 dpi and is normalized against *NbActin* transcripts in the same sample. The data were analyzed using Student's *t‐*test (^*^ *p* < 0.05, ^**^ *p* < 0.01), and asterisks denote significant differences.

We speculated that NbSMG7 and NbUPF3 decreased viral RNA accumulation by NMD to trigger viral RNA degradation. The CGMMV genome RNA harbors one iTC in the RdRP‐L‐encoding region, following a like‐3'UTR (L‐3'UTR). Thus, YFP‐RdRP‐L, YFP‐RdRP‐S, YFP‐MP, and YFP‐CP were co‐expressed with Myc‐GUS (Mock), Myc‐NbSMG7, or Myc‐NbUPF3. The YFP accumulation was analyzed by RT‐qPCR using primers that specifically recognize the YFP sequence presented in these transcripts. As expected, the accumulation of the YFP‐RdRP‐L in the Myc‐NbSMG7 or Myc‐NbUPF3 group was much lower than in the controls (Figure S3, Supporting Information). The expressions of Myc‐GUS, Myc‐NbSMG7, or Myc‐NbUPF3 has no noticeable effects on the transcript accumulations of YFP‐RdRP‐S, YFP‐MP, and YFP‐CP (Figure S3A, Supporting Information). Given that RdRP‐L contains an iTC that NMD might recognize,^[^ ^33^ ^]^ we thus constructed an RdRP‐L mutant (RdRP‐L^miTC^) by the mutation of two bases (3433‐3434 bp: TA to AT) in the iTC position (Figure 2H). To test whether NbSMG7 or NbUPF3 is associated with viral RNA transcripts, we performed RNA immunoprecipitation (RIP) coupled with qRT‐PCR to analyze the wild‐type RdRP‐L and RdRP‐L^miTC^ RNA in the Myc‐NbSMG7‐ and Myc‐NbUPF3‐expressing leaves. As shown in Figure 2I, RdRP‐L rather than RdRP‐L^miTC^ RNA was efficiently immunoprecipitated with anti‐Myc antibodies, which specifically bound to Myc‐NbSMG7 and Myc‐NbUPF3 at 24 hpi, confirming that NbSMG7 and NbUPF3 could bind RdRP‐L effectively in vivo. Furthermore, we also used RT‐qPCR to analyze the degradation of the Myc‐RdRP‐L and Myc‐RdRP‐L^miTC^ transcripts by detecting their RNA accumulation levels in YFP‐NbSMG7‐L5 and YFP‐NbUPF3‐L7 transgenic plants (**Figure** **3**A–C), and the result revealed that accumulation of Myc‐RdRP‐L^miTC^ compared to Myc‐RdRP‐L was almost unaffected in plants expressing YFP‐NbSMG7 or YFP‐NbUPF3 at 60 hpi (Figure 2J). These results demonstrate that NbSMG7 and NbUPF3 could recognize the iTC to mediate the degradation of viral RNA transcripts.

### 2.3 NbSMG7 and NbUPF3 Negatively Regulate CGMMV Infection

We further obtained T2 generation transgenic *N. benthamiana* plants overexpressing YFP‐NbSMG7 or YFP‐NbUPF3, which displayed similar growth prototypes to wild‐type (Wt) *N. benthamiana* plants and accumulated high levels of the YFP‐NbSMG7 protein, or the YFP‐NbUPF3 protein, respectively (Figure 3A–C). After the inoculation of CGMMV infectious clones in these plants, YFP‐NbSMG7 or YFP‐NbUPF3 transgene *N. benthamiana* plants showed much milder mosaic symptoms and accumulated lower levels of viral RNA and CP (Figure 3D,E).

To further investigate the effect of silencing *NMD* factors on CGMMV systemic infection, a modified tobacco rattle virus (TRV)‐based virus‐induced gene silencing (VIGS) vector carrying the partial sequence of GUS (as a control), *NbSMG7*, *NbUPF3*, or *NbSMG7* and *NbUPF3* (*NbSMG7‐NbUPF3*) was pre‐inoculated into *N. benthamiana* plants to knock down the expression of *NbSMG7*, *NbUPF3*, or both *NbSMG7* and *NbUPF3* (Figure 3H,I). Silenced plants at 7 dpi were then inoculated with CGMMV. At 14 dpi, *NbSMG7*, *NbUPF3*, or *NbSMG7‐NbUPF3*‐silenced plants developed severe mosaic symptoms in upper leaves compared to TRV‐GUS‐treated plants (Figure 3J). In agreement with this observation, higher levels of viral RNAs were found in *NbSMG7*, *NbUPF3*, or *NbSMG7‐NbUPF3*‐silenced plants than in control plants at 14 dpi (Figure 3K). It is noteworthy that the silencing of both *NbSMG7* and *NbUPF3* had more pronounced effects on viral symptoms and viral RNA accumulations compared to the silencing of either gene alone (Figure 3J,K), suggesting that NbSMG7 and NbUPF3 cooperatively mediate the NMD pathway to defend against CGMMV infection.

We also constructed transgenic *smg7* and *upf3 N. benthamiana* mutants using the CRISPR/Cas9 technology in this study. Compared to Wt *N. benthamiana* plants, the *smg7* and *upf3* mutants showed obvious developmental defects, including dwarfing in their later growth stages and infertility (Figure S3, Supporting Information). Thus, we speculate that these two genes in *N. benthamiana* may also be involved in plant reproduction, just like their orthologs in *Arabidopsis*.^[^ ^36^, ^37^, ^38^, ^39^, ^40^ ^]^ Therefore, due to the infertility of *smg7* and *upf3 N. benthamiana* mutants, we failed to get their offspring seeds; thus, we could not analyze the virus infection in these mutants further.

### 2.4 CGMMV Triggers the Autophagy Pathway to Degrade NbSMG7

**Figure 4:**
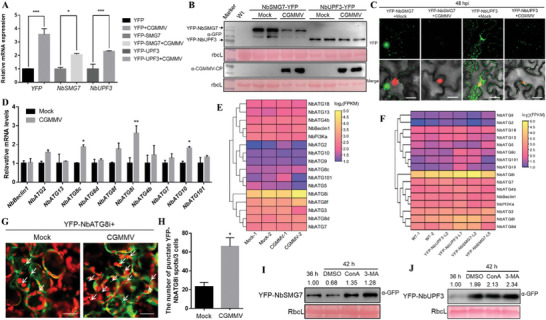
The effects of CGMMV on the RNA and protein accumulation of YFP, YFP‐NbSMG7, or YFP‐NbUPF3. A,C,D) Wild type or RFP‐H2B transgene *N. benthamiana* leaves were infiltrated with *Agrobacterium* cultures to express YFP, YFP‐NbSMG7, or YFP‐NbUPF3 alone or together with CGMMV. Total RNA and proteins were extracted from the infiltrated zones at 48 hpi. For RT‐qPCR analysis (A), the values of YFP, YFP‐NbSMG7, and YFP‐NbUPF3 at 48 hpi were normalized against *NbActin* transcripts in the same sample. For immunoblotting analysis B), total proteins were detected with anti‐GFP antibodies. For confocal observations (C), the infiltrated RFP‐H2B transgene *N. benthamiana* leaves were examined by confocal microscopy at 48 hpi. Three independent infiltrations repeated these experiments three times, and >20 cells per sample were observed in each replicate; representative results are shown. Scale bar: 25 µm. (D) Effects of CGMMV infection on the expression of ATGs at 12 dpi. *N. benthamiana* leaves were agroinfiltrated with Mock and CGMMV. Total RNAs were extracted from infiltrated zones at 12 dpi. E) Transcriptome analysis of DEGs in *N. benthamiana* infiltrated with Mock or CGMMV. Hierarchical clustering of DEGs based on log10 FPKM value. The color (from blue to yellow) represents gene expression intensity (log10 FPKM) from low to high. F) Transcriptome analysis of DEGs in wild type and YFP‐NbSMG7/YFP‐NbUPF3 overexpression transgene *N. benthamiana*. Hierarchical clustering of DEGs based on log10 FPKM value. The color (from blue to yellow) represents gene expression intensity (log10 FPKM) from low to high. G) Confocal micrographs showing *N. benthamiana* leaf cells co‐infiltrated with YFP‐NbATG8i and Mock or CGMMV infectious clone (CGMMV) at 60 hpi. Scale bar: 50 µm. H) The average number of YFP‐NbATG8i spots per 3 cells. Infiltration experiments were repeated three times, and 30 cells were counted for the punctate spots. The average number was calculated using 3 cells as a unit. Values represent the mean spot ±standard deviation (SD) per 3 cells. I,J) The effect of the autophagy inhibitor 3‐MA and Con A on the accumulation of YFP‐NbSMG7 and YFP‐NbUPF3. Immunoblotting was performed using anti‐GFP antibodies. Statistical analysis was performed using Student's *t*‐test (two‐sided, ^*^ *p* < 0.05, ^**^ *p* < 0.01, ^***^ *p *< 0.001) (A,D). All immunoblotting assays in these Figures were repeated at least three times, and each experimental sample was derived from three individual infiltrations. One representative immunoblotting is shown. The RbcL was stained with Ponceau S to indicate equal protein loading (B,I,J).

To help understand whether and how CGGMV infection affects the NMD pathway, we analyzed the effects of CGMMV infection on the RNA and protein accumulations of NbSMG7 and NbUPF3. YFP, YFP‐NbSMG7and YFP‐NbUPF3 were infiltrated into *N. benthamiana* leaves alone or together with and the infectious clone of CGMMV. The infiltrated leaves at 48 hpi were used for RNA and protein extraction and confocal observations. As shown in **Figure** **4**A, the RNA levels of YFP, YFP‐NbSMG7, and YFP‐NbUPF3 in CGMMV‐infiltrated leaves increased significantly compared to when they were expressed alone, which might be caused by virus‐induced RNA silencing inhibition. However, the co‐expression of CGMMV decreased the fluorescence signals and protein accumulations of YFP‐NbSMG7 and YFP‐NbUPF3 (Figure 4B,C), while it increased the fluorescence signals and protein accumulation of YFP (Figure S5, Supporting Information).

To examine whether CGMMV infection induces autophagy, leading to the decreased protein accumulation of YFP‐NbSMG7 and YFP‐NbUPF3, the expression levels of ATGs were analyzed in Mock, and CGMMV‐infiltrated *N. benthamiana* leaves at 3 dpi. As shown in Figure 4D, the expression of several ATGs, including *NbATG8i*, was upregulated by CGMMV infection, indicating that CGMMV infection might trigger the autophagy pathway. We further constructed RNA‐seq libraries of Mock and CGMMV‐infected *N. benthamiana* plants at 12 dpi, and Wt, YFP‐NbSMG7 and YFP‐NbUPF3 transgene *N. benthamiana* after seeding 20 days (Figure S6, Supporting Information). We found that transgenic overexpression of NbSMG7 and NbUPF3 and CGMMV infection could upregulate the expression of many *ATGs* (Figure 4E,F), indicating the autophagy pathway was activated in the high expression of NMD factors or the invasion of CGMMV. In addition, the increased YFP‐NbATG8i granules in CGMMV‐infiltrated *N. benthamian*a leaves compared to Mock‐infiltrated ones also confirmed that the infection of CGMMV triggered the autophagy pathway (Figure 4G,H; Figure S7, Supporting Information).

To determine whether the decreased protein accumulation of YFP‐NbSMG7 or YFP‐NbUPF3 in CGMMV infection can be attributed to autophagic degradation, the sensitivity of YFP‐NbSMG7 or YFP‐NbUPF3 to autophagy inhibitors, 3‐methyladenine (3‐MA) that inhibits the initiation of autophagy, and concanamycin A (Con A) that is the specific vacuolar ATPase inhibitor, was tested. *N. benthamiana* leaves were agroinfiltrated with a vector to express YFP‐NbSMG7 or YFP‐NbUPF3 followed by infiltration of DMSO (control), 3‐MA (5 mm), or Con A (1 µm ConA) after 36 hpi. Samples were collected from leaves after an additional 6 h incubation for confocal and immunoblotting assay. The treatment with 3‐MA and ConA led to a more vigorous fluorescence intensity of YFP‐NbSMG7 rather than YFP‐NbUPF3 at 42 hpi, compared to the DMSO treatment (Figure S6A, Supporting Information), indicating that the autophagy pathway is probably involved in the degradation of YFP‐NbSMG7. Supporting this conclusion, YFP‐NbSMG7 rather than YFP‐NbUPF3 accumulated higher levels in 3‐MA or ConA‐treated leaves than in DMSO‐treated leaves (Figure 4I,J). However, 3‐MA or ConA did not have a noticeable impact on the RNA accumulation of YFP‐NbSMG7 and YFP‐NbUPF3 (Figure S6B,C, Supporting Information).

### 2.5 NbATG8i Interacts with NbSMG7 to Mediate its Autophagic Degradation

**Figure 5:**
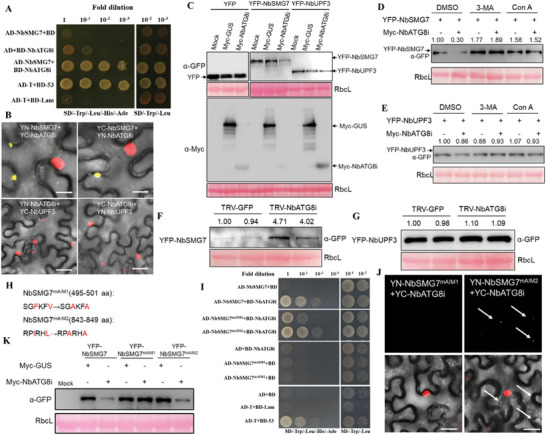
NbATG8i interacts with NbSMG7 to mediate its degradation, and the AIM1 site is required for degradation. A) Y2H assays for the interaction between NbATG8i and NbSMG7. NbSMG7 and NbATG8i were fused to AD and BD vectors, respectively. Y2H Gold yeast cells co‐transformed with the indicated plasmids were subjected to 10‐fold serial dilutions and plated on SD/‐Trp, ‐Leu, ‐His medium. Yeast cells co‐transformed with AD‐NbSMG7 and the empty BD, or with the empty AD and BD‐NbATG8i, or with AD‐T and BD‐Lam are negative controls, and yeast cells co‐transformed with AD‐T and BD‐53 are positive controls. B) BiFC assays between NbATG8i and NbSMG7 in the leaves of RFP‐H2B transgenic *N. benthamiana* plants. RFP‐H2B transgenic leaves were co‐infiltrated with agrobacterium cultures harboring the following pairs of plasmids: YN‐NbATG8i and YC‐NbSMG7 (Lane I), YC‐NbATG8i and YN‐NbSMG7 (Lane II), YN‐NbATG8i and YC‐P3N‐PIPO (Lane III, negative control), YC‐NbATG8i and YN‐P3N‐PIPO (Lane IV, negative control). Confocal imaging was performed at 48 hpi. Bars, 25 µm. C) Overexpression of NbATG8i decreased the protein accumulation of YFP‐NbSMG7 but not YFP‐NbUPF3. Immunoblotting of total protein extracts from the *N. benthamiana* leaves agroinfiltrated with Myc‐GUS (‐) or the plasmids indicated. Immunoblotting was performed using anti‐GFP and anti‐Myc antibodies. D,E) The autophagy inhibitor 3‐MA or Con A affects the protein accumulation of YFP‐NbSMG7 and YFP‐NbUPF3. Total proteins were extracted from *N. benthamiana* leaves agroinfiltrated with the indicated plasmids, followed by treatment with DMSO (control), 3‐MA, or Con A (10 um) for 9 h. F,G) The effect of the silencing of *NbATG8i* on the protein accumulation of YFP‐NbSMG7 and YFP‐NbUPF3. *N. benthamiana* plants were pre‐treated with relevant TRV‐recombinant vectors for 14 days and then infiltrated with YFP‐NbSMG7 or YFP‐NbUPF3. Immunoblotting was performed with anti‐GFP and anti‐Myc antibodies. Each experiment was repeated at least three times. H) Schematic diagram on the mutation of NbSMG7 AIMs. I) Y2H assays for the interaction between NbATG8i and NbSMG7, NbSMG7^mAIM1^, and NbSMG7^mAIM2^. Y2H Gold yeast cells co‐transformed with the indicated plasmids were subjected to 10‐fold serial dilutions and plated on SD/‐Trp, ‐Leu, ‐His medium. J) BiFC assays between NbATG8i and NbSMG7^mAIM1^ or NbSMG7^mAIM2^ in the leaves of RFP‐H2B transgenic *N. benthamiana* plants. Confocal imaging was performed at 48 hpi. Bars, 50 µm. K) Overexpression of NbATG8i decreased the protein accumulation of YFP‐NbSMG7 and YFP‐NbSMG7^mAIM2^. Immunoblotting of total protein extracts from the *N. benthamiana* leaves agroinfiltrated with Myc‐GUS or the plasmids indicated. Immunoblotting was performed using anti‐GFP antibodies. Each experiment was repeated at least three times. The RbcL was stained with Ponceau S to indicate equal protein loading (C–G,K).

To further address whether NbSMG7 and NbUPF3 were degraded via autophagy and, if so, to determine which ATG was involved in this process, the interaction between NbSMG7, or NbUPF3 and NbBeclin1, NbPI3K, NbATG5, NbATG7, NbATG8a, NbATG8c1, NbATG8c2, NbATG8c3, and NbATG8i was screened by Y2H assays. As shown in **Figure** **5**A and Table S2 (Supporting Information), a strong interaction between NbSMG7 and NbATG8i was found. No interaction was found between NbUPF3 and any ATGs (Table S2, Supporting Information). BiFC confirmed the interaction between NbSMG7 and NbATG8i in RFP‐H2B transgenic *N. benthamiana* plants, and their interaction complex was localized in the cytoplasm, forming irregular granules (Figure 5B). No interaction fluorescence was observed when YN‐NbATG8i and YC‐NbUPF3, or YC‐NbATG8i and YN‐NbUPF3 were co‐infiltrated in RFP‐H2B transgenic *N. benthamiana* plants. Furthermore, the NbATG8i‐mediated degradation of NbSMG7 was noticeable when the two proteins were co‐expressed together (Figure 5C). This degradation could be blocked by the treatment of 3‐MA or Con A or the knockdown of NbATG8i (Figure 5D–G). However, we did not get similar results on NbUPF3 (Figure 5C–G), indicating that NbUPF3 might not be degraded by autophagy.

Two ATG8‐interacting motifs (AIMs) that matched the consensus amino acid sequence X[DE][DE][WFY] [ADCQEIGNLMFPS TWYV]X[LIV] were identified in NbSMG7: SGFKFV and RPIRHL. Mutations in the AIM motif of NbSMG7 were generated by replacing SGFKFV with SGAKFA (NbSMG7^mAIM1^) and RPIRHL by RPARHA (NbSMG7^mAIM2^) using overlapping PCR primers (Figure 5H; Table S1, Supporting Information). By Y2H and BiFC assays, we found that the mutation in the AIM1 of NbSMG7 abolished its interaction with NbATG8i, but the mutation in the AIM2 of NbSMG7 did not affect its ability to interact with NbATG8i (Figure 5I,J). In addition, overexpression of NbATG8i decreased the protein accumulation of YFP‐NbSMG7 and YFP‐NbSMG7m^AIM2^ but was unable to decrease the protein accumulation of YFP‐NbSMG7^mAIM1^ (Figure 5K). These results indicate that the autophagy‐related protein NbATG8i interacted with NbSMG7 via its AIM1 to mediate its autophagic degradation.

### 2.6 NbATG8i Plays an Anti‐viral Role, and Knockdown of *NbATG8i* and *NbSMG7* or *NbATG8i* and *NbUPF3* Synergistically Enhances CGMMV Infection

**Figure 6:**
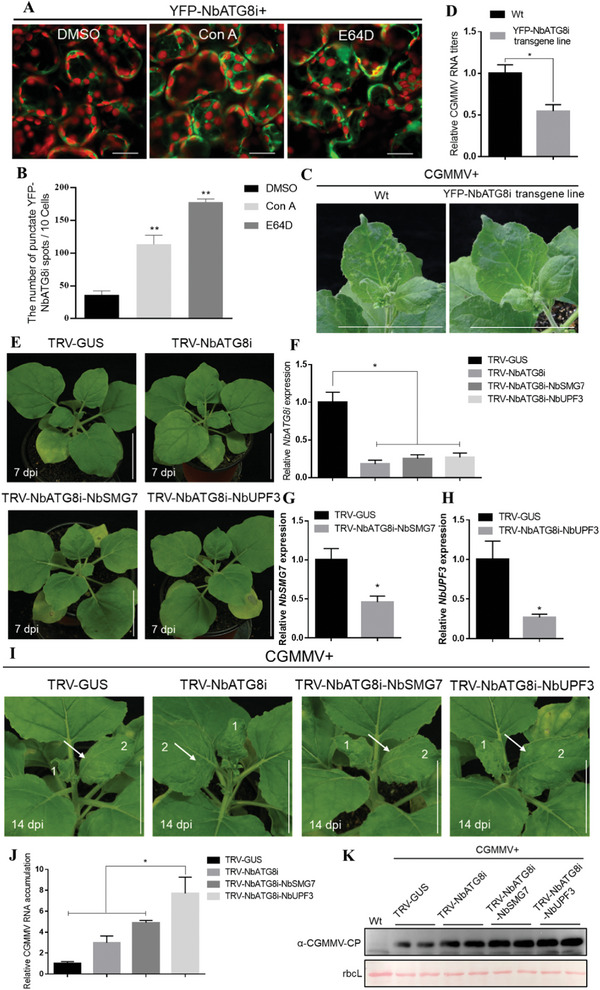
NbATG8i and NMD factors synergistically defend against CGMMV infection. A)YFP‐NbATG8i infiltrated leaves were treated with DMSO, concanamycin A (Con A), or E64D after 36 hpi and confocal images were taken at 6 h after treatment. Bars, 25 µm. B) The average number of YFP‐NbATG8i spots per 10 cells. Infiltration experiments were repeated three times, and 60 cells were counted for the punctate spots. The average number was calculated using 10 cells as a unit. Values represent the mean spot ±standard deviation (SD) per 10 cells. C) CGMMV symptoms in Wt and YFP‐NbATG8i transgene *N. benthamiana* plants inoculated with CGMMV infectious clones at 14 dpi. Bars, 5 cm. D) RT‐qPCR analysis of CGMMV RNA levels. RNA was extracted from the leaves in (C) at 14 dpi. E) Phenotypes of TRV‐GUS, TRV‐NbATG8i, TRV‐NbATG8i‐NbSMG7, or TRV‐NbATG8i‐NbUPF3 treated plants at 14 dpi. Bars, 10 cm. F–H) Confirmation of knockdown of *NbATG8i*, *NbATG8i*, and *NbSMG7*, or *NbATG8i* and *NbUPF3* in newly developing leaves of *N. benthamiana* plants at 14 dpi (A) by RT‐qPCR. I) CGMMV symptoms in plants pre‐inoculated with TRV1 together with TRV2‐GUS, TRV‐NbATG8i, TRV‐NbATG8i‐NbSMG7, or TRV‐NbATG8i‐NbUPF3 for 7 days and then infected by CGMMV infectious clones. Plants were photographed at 14 dpi. J) Quantification of CGMMV genomic RNA in the above plants. RNA was extracted from the systemically infected leaves at 14 dpi. The values are presented as means ± SD relative to CGMMV‐inoculated TRV‐GUS‐treated leaves at 14 dpi and are normalized against *NbActin* transcripts in the same sample. The data were analyzed using Student's *t*‐test, and asterisks denote significant differences (^*^ *p* < 0.05, ^**^ *p* < 0.01). K) Immunoblotting of total protein extracts from (E). Immunoblotting was performed using anti‐CGMMV CP antibodies. Each experiment was repeated at least three times. The rbcL was stained with Ponceau S to indicate equal protein loading.

**Figure 7:**
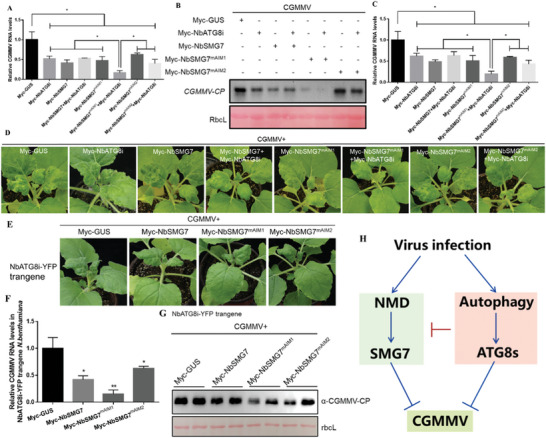
Overexpression of NbATG8i impairs the NbSMG7‐mediated inhibition of CGMMV infection. A) RT‐qPCR analysis of CGMMV RNA levels. RNA was extracted from leaves agroinfiltrated with CGMMV infectious clones and the indicated plasmids at 60 hpi. Values represent means ± SD relative to plants infiltrated with CGMMV infectious clones and Myc‐GUS. B) Immunoblotting of total protein extracts from the *N. benthamiana* leaves agroinfiltrated with CGMMV infectious clones and the indicated plasmids at 60 hpi. Immunoblotting was performed using anti‐CP and anti‐Myc antibodies. Each experiment was repeated at least three times. The rbcL was stained with Ponceau S to indicate equal protein loading. C,D) Symptoms and viral RNA accumulations of CGMMV infectious clones together with the indicated plasmids‐infected plants at 14 dpi. Values represent means ± SD relative to plants infected with CGMMV and GUS. Statistical significance (^*^ *p* < 0.05, ^**^ *p* < 0.01) was shown by Student's *t*‐test (A,D). E) Symptoms of NbATG8i‐YFP transgene *N. benthamiana* leaves agroinfiltrated with CGMMV infectious clones and overexpressing NbSMG7, NbSMG7^mAIM1^, NbSMG7^mAIM2^ as the indicated plasmids at 60 hpi. F) RT‐qPCR analysis of CGMMV RNA levels. RNA was extracted from the leaves in (E) at 60 hpi. Values represent means ± SD relative to plants infiltrated with CGMMV infectious clones and Myc‐GUS. G) Immunoblotting of total protein extracts from the leaves in (E) at 60 hpi. Immunoblotting was performed using anti‐CP and anti‐Myc antibodies. Each experiment was repeated at least three times. The rbcL was stained with Ponceau S to indicate equal protein loading. H) A proposed model of the crosstalk of NMD and autophagy during virus infection.

Expression of YFP‐NbATG8i in *N. benthamiana* leaves can accumulate more autophagy granules and YFP‐NbATG8i proteins after treatment with autophagy inhibitors, including 3‐methyladenine (3‐MA), concanamycin A (Con A), and E64D (**Figure** **6**A,B; Figure S7A,B, Supporting Information), pointing that NbATG8i can induce autophagy. Our previous study has shown that the autophagy core component, Beclin1, restricts CGMMV infection. We further found that YFP‐NbATG8i transgenic plants, compared to Wt *N. benthamiana* plants, displayed more resistance to CGMMV infection, which was accompanied by milder virus symptoms and less viral RNA and protein accumulation (Figure 7C,D), supporting that autophagy and NbATG8i restrict viral infection.

To further investigate the crosstalk between autophagy and NMD in viral infection, we used TRV‐VIGS to silence *NbATG8i*, *NbATG8i* and *NbSMG7*, or *NbATG8i* and *NbUPF3* (Figure 6A–D). As expected, *NbATG8i*, *NbATG8i* and *NbSMG7*, or *NbATG8i* and *NbUPF3*‐silenced plants developed more severe viral symptoms in upper leaves compared to TRV‐GUS‐treated plants (Figure 6E). In agreement with this observation, higher levels of viral RNAs were found in *NbATG8i*, *NbATG8i‐NbSMG7*, or *NbATG8i‐NbUPF3*‐silenced plants than in control plants at 14 dpi (Figure 6F). Of note, the silencing of *NbATG8i* and *NbSMG7* together, or *NbATG8i* and *NbUPF3* together, compared to the individual silencing of *NbATG8i* resulted in more several viral symptoms and more viral RNA accumulations (Figure 6E,F), supporting that NMD and autophagy function as distinct branches in *N. benthamiana* defenses against CGMMV infection.

### 2.7 Overexpression of NbATG8i Impairs the NbSMG7‐Mediated Viral Suppression

The above data showed that both autophagy and NMD play anti‐viral roles, but autophagy can degrade the key NMD effector, NbSMG7. To further untangle the complexities of these two pathways in the context of virus infection, we infiltrated CGMMV together with Myc‐tagged GUS, NbATG8i, NbSMG7, NbSMG7^mAIM1^ (no interaction with ATG8i), NbSMG7^mAIM2^ (interaction with ATG8i), NbATG8i and NbSMG7, NbATG8i and NbSMG7^mAIM1^, or NbATG8i and NbSMG7^mAIM2^ into *N. benthamiana* leaves. At 60 hpi, overexpression of all constructs inhibited CGMMV RNA and protein levels compared to Myc‐GUS (**Figure** **7**A,B). Of note, overexpressing NbATG8i and NbSMG7^mAIM1^ led to much lower RNA levels of CGMMV compared to overexpressing NbATG8i, NbSMG7^mAIM1^, NbSMG7^mAIM2^, NbATG8i, and NbSMG7, or NbATG8i and NbSMG7^mAIM2^ (Figure 7A,B). The above‐infiltrated plants were maintained to monitor symptom development and analyze viral RNA accumulation in newly emerged leaves at 14 dpi. Similarly, the overexpression of NbATG8i, NbSMG7^mAIM1^, NbSMG7^mAIM2^, NbATG8i and NbSMG7, NbATG8i and NbSMG7^mAIM1^, or NbATG8i and NbSMG7^mAIM2^ compared to GUS also attenuated viral symptoms and systemic infection and decreased viral RNA accumulations (Figure 7C,D). It is worth noting that the accumulations of CGMMV RNA in the plants co‐infiltrated with NbATG8i and NbSMG7^mAIM1^ were lower than in the plants infiltrated with NbATG8i or NbSMG7^mAIM1^ alone (Figure 7D).

Furthermore, using NbATG8i transgenic *N. benthamiana* plants, we found a similar result by overexpressing NbSMG7, NbSMG7^mAIM1^ (no interaction with ATG8i), or NbSMG7^mAIM2^ (interaction with ATG8i) (Figure 7E–G). Therefore, we conclude that the simultaneous overexpression of NbATG8i and NbSMG7 compromised NbSMG7‐mediated suppression of CGMMV infection. These data indicate that the active autophagy pathway could perturb the NMD pathway‐mediated restriction to CGMMV during virus infection, which occurs by NbATG8i‐mediated degradation of NbSMG7 via its AIM1.

## 3 Discussion

All eukaryotes are equipped with the RQC system that consists of at least three pathways, i.e., NMD, NSD, and NGD, to monitor the quality of mRNAs during translation. It is widely accepted that dysfunctional endogenous transcripts are degraded by RQC‐dependent exonucleolytic decay. Exogenous and selected endogenous RNAs are eliminated through RNA silencing.^[^ ^41^ ^]^ RNA silencing is one of the most critical immune defenses against invasive viruses by targeting and degrading viral RNAs in plants.^[^ ^42^, ^43^ ^]^ Other antiviral defense mechanisms targeting RNA have recently been identified, including RNA decay, RQC, and m^6^A RNA modifications, which greatly expand plant interactions with viral RNAs.^[^ ^44^ ^]^ Here, we investigated the biological functions of NbSMG7 and NbUPF3 and found that both NbSMG7 and NbUPF3 mainly formed small bright granules in the cytoplasm, and NbSMG7 rather than NbUPF3 co‐localized with the P‐body marker, DCP1‐CFP1 (Figure 1), supporting that NMD is coupled with RNA processing and decay. It has been shown that RNA silencing was impaired in Arabidopsis *upf1* mutant, indicating a potential connection between NMD and RNA silencing in plants.^[^ ^37^ ^]^ However, no RNA silencing activation or suppression was found when GFP was co‐expressed with NbSMG7 or NbUPF3 in 16c transgenic *N. benthamiana* plants (Figure S1, Supporting Information), indicating that NbSMG7 and NbUPF3 have no prominent regulation roles in silencing exogenous GFP transcripts. This observation aligns with studies in *Caenorhabditis elegans*, where several NMD factors were found to be unnecessary for RNA silencing, highlighting the independence of the NMD and RNA silencing pathways.^[^ ^35^ ^]^ In addition, no noticeable RNA differences were detected when the virus construct TuMV‐GFP, and several GFP reporter constructs without PTCs were co‐expressed with UPF1 or its dominant mutant, supporting that the NMD factors target its selected substrates rather than any other redundant RNAs. Of note, the NMD activity can be regulated by several types of intracellular disturbances, including promoting apoptosis by upregulation of genes involved in apoptotic stress response, impairment of NMD response by caspase cleavage UPF1 and UPF2, inhibition of translation initiation, down‐regulation expression of core NMD factors,^[^ ^45^ ^]^ indicating a broader role of NMD in gene expression regulation. Supporting this, NMD is also one of the main regulatory processes through which pattern recognition receptors (PRRs) fine‐tune resistance gene (*R*) transcript levels to reduce fitness costs and achieve effective immunity.^[^ ^46^ ^]^ Jung et al. found that PRR recognition of microbe‐associated molecular patterns (MAMPs) controlled the levels of *R* transcripts carrying NMD‐sensitive signatures by inducing UPF1/UPF3 ubiquitination. They revealed that Arabidopsis UPF1 and UPF3 were destroyed via ubiquitination at an early stage of *PstDC3000* infection, which led to the steady‐state levels and the stability of *R* transcripts in bacterial‐infected plants,^[^ ^45^ ^]^ pointing to an additional layer of plasticity between the PRRs‐ and R‐induced immunity by NMD.

*Arabidopsis* plants with knockout of *UPF1*, *UPF2*, *UPF3*, or *SMG7* display a range of developmental phenotypes that depend on its residual expression or its mutation position, and these phenotypic defects include plant dwarfing, narrower and more jagged rosette leaves, aberrant flower formation, seedling lethality infertility.^[^ ^36^, ^37^, ^38^, ^39^, ^40^ ^]^ For example, the weakest allele, *upf1‐4*, displays a very subtle phenotype, having smaller and slightly indented leaves. *Upf1‐5* shows floral defects in some flowers and has late‐flowering and leaf‐indentation phenotypes; *upf1‐3* shows seeding lethality after 12 days. *Upf3 *alleles also display defective vegetative and floral phenotypes.^[^ ^37^ ^]^ *smg7* mutants (*smg7‐1, 3*) with insertions in the central region of SMG7 are viable but show retarded growth and are infertile due to abortive meiosis; *smg7‐5* with the disruption in its most N‐terminal display embryonic lethality.^[^ ^40^, ^47^, ^48^ ^]^ In this study, we obtained T0 transgenic *smg7* and *upf3 N. benthamiana* mutants using CRISPR/Cas9 and found significant development defects (Figure S4, Supporting Information). We failed to collect its seeds further due to the infertility of these mutants. These data together indicate that certain expressions of SMG7 and UPF3 activity are essential for plant viability. However, SMG7 and UPF3 are crucial factors in the NMD core machinery and could also be involved in other cellular processes.

Increasing evidence has shown that NMD, NGD, and NSD serve as common virus restriction strategies by recognizing virus sequences with iTC, with long 3' UTRs, or with specific motifs in plants.^[^ ^11^, ^15^, ^34^ ^]^ NMD removes the mRNA species containing PTCs or long 3' UTRs.^[^ ^49^ ^]^ RNA viruses often contain internal iTCs to achieve the maximization of coding ability. Thus, NMD is recognized as a general virus resistance mechanism in plants and mammals.^[^ ^11^, ^12^, ^34^ ^]^ Consistent with this, we found that NbSMG7 and NbUPF3, the essential components of NMD, negatively regulated CGMMV infection by targeting virus iTC for degradation to limit virus infection (Figure 3), supporting the anti‐viral role of NMD in virus infection. Notably, many viruses with high pathogenic ability could inhibit or even evade NMD in animals and plants.^[^ ^6^, ^48^, ^50^ ^]^ For example, in plants, potyviruses employ the translation strategy of a polyprotein using the long mRNA to escape from NMD‐mediated virus restriction;^[^ ^6^, ^11^ ^]^ the transactivator protein encoded by CaMV can inhibit NMD by targeting VARICOSE, a scaffold protein of the decapping complex.^[^ ^51^ ^]^ The pea enation mosaic virus 2 p26 protein, a long‐distance viral movement protein, could confer protection for both cellular and viral transcripts from NMD for more favorable infection through an unknown mechanism.^[^ ^52^ ^]^ The host NMD response reduces viral infection, which in turn is counteracted by viral regulation of NMD, and it appears to be an evolutionarily conserved relationship. However, since viruses can efficiently counter‐defend ‐NMD and other surveillance systems so these covered underlying mechanisms can be particularly difficult to uncover.

Autophagy is a critical anti‐viral pathway in plants. It is known that several ATGs act as cargo receptors or autophagy regulators to selectively interact with viral effectors to target them for autophagic degradation. For example, ATG8f interacts with CLCuMuV βC1;^[^ ^23^ ^]^ ATG6 interacts with TuMV NIb, CGMMV RdRP and PepMV RdRp;^[^ ^25^ ^]^ ATG8i interacts with the C1 protein of TLCYnV.^[^ ^6^ ^]^ In this study, we found that viral infection decreased the protein accumulation of SMG7 (Figure 4B; Figure S9, Supporting Information). ee screened the potential interactions between ATGs and SMG7, or ATGs and UPF3 of *N. benthamiana*, and found that NbATG8i interacted with the NMD factor, NbSMG7 (Figure 5A). We further demonstrated that the AIM1 motif of NbSMG7 was required for its interaction with NbATG8i (Figure 5H–K), which guided its autophagic degradation and impaired the SMG7‐mediated virus restriction during CGMMV infection (Figure 5D,E). Although multiple strategies employed by RNA viruses for evasion or subversion of NMD have been reported,^[^ ^11^, ^34^, ^49^ ^]^ the promotion of the autophagic degradation of NMD factors by virus infection has not been clearly elucidated. As seen in Figure S10 (Supporting Information), we found more expression levels of RdRP‐L but not RdRP‐L^miTC^ in Wt plants rather than in YFP‐ATG8i transgenic plants. This result is consistent with our previous finding, supporting that NbATG8i negatively regulates SMG7 in YFP‐NbATG8i overexpression lines. The excessive activation of NMD can be detrimental to some biological processes. SMG7 itself contains a long 3' UTR structure, which may induce NMD by a negative feedback. Similarly, UPF3 transcript levels are critical for proper NMD regulation through its delicate balance of negative feedback loops and transcriptional restriction in Arabidopsis.^[^ ^53^ ^]^ Thus, the autophagic degradation of NMD factors might be an inner balanced and protective mechanism to coordinate resistance and other physiologic pathways in plants. Moreover, our study suggests the existence of an unknown mechanism for degrading UPF3 in virus‐infected plants (Figure 4B,C), implying the intricacies involved in NMD‐virus interactions. Taken together, we demonstrate that both NMD and autophagy are antiviral pathways. However, NbATG8i‐mediated the autophagic degradation of NbSMG7 compromised their combined anti‐viral impact (Figure 6). The NbSMG7 degradation by autophagy may be an additional way by which the cell regulates the NMD efficiency (Figure S9, Supporting Information), and, interestingly, this mode of regulation has an impact on viral defense. Therefore, this study provides a novel role of autophagy in eukaryotes and reveals the crosstalk and feedback of NMD and autophagy in the context of virus infection, which provides new insight into the understanding of complex plant and microbe interactions and the crosstalk of different plant defense pathways.

## 4 Experimental Section

### Plant Materials and Growth Conditions

Wild‐type *N. benthamiana*, transgenic *N. benthamiana* lines expressing GFP (line 16c), YFP‐NbSMG7, YFP‐NbUPF3, RFP‐H2B, and transgenic NbSMG7‐Cas9 and NbUPF3‐Cas9 *N. benthamiana* seedlings were potted in soil and placed in an insect‐free growth chamber under controlled conditions with 60% relative humidity and a 16 h: 8 h, light: dark, 25°:18 °C regime.

### Plasmid Construction

Sequence data from this article can be found in the GenBank data library under the following accession number.: NbSMG7 (MW343792), NbUPF3 (MW380239), NbATG8i (KX369401). NbSMG7, NbUPF3, and NbATG8i coding sequences were obtained from complementary DNA (cDNA) synthesized from *N. benthamiana* leaves. The full‐length RdRPL, RdRPS, MP, and CP sequences were cloned from the CGMMV genome. Gateway technology was used to generate all constructs for yeast two‐hybrid (Y2H) and for transient expression in planta used in this study unless stated otherwise. Briefly, the resulting DNA fragments from PCR were purified and cloned into the entry vector pDONR221 (Invitrogen) by recombination using BP Clonase (Invitrogen). Insertions in the resulting pDONR clones were verified by DNA sequencing. Then the linearized intermediate pDONR221 plasmids carrying inserts were further transferred into Gateway‐compatible vectors: pGADT7‐DEST, pGBKT7‐DEST, pEarleygate101, pEarleygate102, pEarleygate103, pEarleygate104, 35S‐YN gateway, 35S‐YC gateway, pEarleygate104, or Myc‐Flag‐Myc4 vector to generate the corresponding constructs. To construct a tobacco rattle virus (TRV)‐based recombinant virus‐induced gene silencing (VIGS) vector containing *NbSMG7*, *NbUPF3*, or *NbATG8i*, a partial fragment of each gene was generated by PCR amplification using the corresponding primers (Table S1, Supporting Information) and then cloned into the pTRV2 vector using the restriction enzyme sites listed in Table S1 (Supporting Information). For the generation of TRV‐NbSMG7‐NbUPF3, TRV‐NbSMG7‐NbAT8i, TRV‐NbUPF3‐NbAT8i recombinant plasmids, the partial sequences of NbUPF3 and NbATG8i were cloned into TRV‐NbSMG7 and TRV‐NbUPF3 vectors using the corresponding restriction enzyme sites listed in Table S1 (Supporting Information). Mutants of NbSMG7 were generated by overlapping PCR using the specific primer pairs given in Table S1 (Supporting Information) and cloned into the gateway‐compatible vectors described above.

### Yeast Two‐Hybrid, Bimolecular Fluorescence Complementation, and Subcellular Localization

For Y2H assays, experiments were performed as described in the Clontech yeast protocol handbook. Briefly, pGADT7‐DEST, and pGBKT7‐DEST recombinant constructs containing the genes of interest were co‐transformed into yeast cells (strain Y2H Gold) and then were plated onto a selective medium lacking tryptophan and leucine (synthetic dextrose (SD)/Trp/Leu) to select co‐transformants. A high‐stringency particular medium lacking tryptophan, leucine, histidine, and adenine (SD/Trp/Leu/His/Ade) to test the interaction. Bimolecular fluorescence complementation (BiFC) and subcellular localization experiments were performed as described previously.^[^ ^25^ ^]^ Genes of interest were transiently expressed by agroinfiltration in wild‐type and transgenic RFP‐H2B *N. benthamiana* leaves. At 36–72 h after infiltration, fluorescence was examined by confocal microscopy (Carl Zeiss laser confocal microscope 980, Germany). The fluorescence parameters were set as described previously.^[^ ^25^ ^]^ The sequential scanning mode was applied for the co‐imaging of different fluorescent proteins. Collected images were analyzed with the Carl Zeiss Application Suite for Advanced Fluorescence software.

### RNA Extraction, Quantitative Reverse Transcription PCR Analysis, and RIP‐qRT‐PCR

Total RNA was isolated from the infiltrated or virus‐infected leaves, as indicated in the manuscript, at different time points using the Trizol reagent. Quantitative reverse transcription PCR (RT‐qPCR) analysis was conducted and analyzed as described previously.^[^ ^25^, ^54^, ^55^ ^]^ *NbActin* was used as an internal control for *N. benthamiana*. A list of primers used in this study is provided in Table S1 (Supporting Information). RIP‐qRT‐PCR was performed as previously described with minor modifications,^[^ ^15^ ^]^ and anti‐Myc antibodies were used specifically bound to Myc‐NbSMG7 and Myc‐NbUPF3 in this experiment.

### Chemical Treatments and Immunoblotting

Infiltration buffer containing dimethyl sulfoxide (DMSO; control) or an equal volume of DMSO with 5 mm 3‐methyladenine (3‐MA) or 1 µm concanamycin A (Con A) for inhibition of autophagy, or an equal volume of DMSO with 100 µm MG132 (a proteasome inhibitor; Sigma–Aldrich) for inhibition of the 26S proteasome, was infiltrated into leaves 6 h before samples were collected for Immunoblotting analysis. Total protein was extracted from infiltrated leaf patches, or systemically infected *N. benthamiana* leaves as described previously.^[^ ^25^, ^55^ ^]^ Immunoblotting was performed with primary mouse polyclonal antibodies: anti‐GFP polyclonal antibodies (1:5000) and anti‐Myc polyclonal antibodies (1:5000), followed with goat anti‐mouse (1:5000) secondary antibodies conjugated to horseradish peroxidase (Abcam, Cambridge, MA, USA). Blotted membranes were washed thoroughly and visualized using chemiluminescence according to the manufacturer's protocol.

## Conflict of Interest

The authors declare no conflict of interest.

## Author Contributions

F.L. designed the project; Y.C. and F.L. carried out experiments with assistance from M.J., L.G., Z.L., and H.H.; all authors analyzed and discussed the data; F.L. and Y.C. wrote the manuscript; all the authors reviewed and approved the manuscript.

## Supporting information

Supporting Information

## Data Availability

The data that support the findings of this study are available from the corresponding author upon reasonable request.
