# Supplementary material for: A Negative Feedback Loop Compromises NMD‐Mediated Virus Restriction by the Autophagy Pathway in Plants
Source: Adv Sci (Weinh). 2024 Jun 21;11(32):2400978. doi: 10.1002/advs.202400978 (PMC11348178; doi:10.1002/advs.202400978)
Supplement: Supplementary file 1 — Supporting Information [file ADVS-11-2400978-s001.docx]

**Supporting Information**


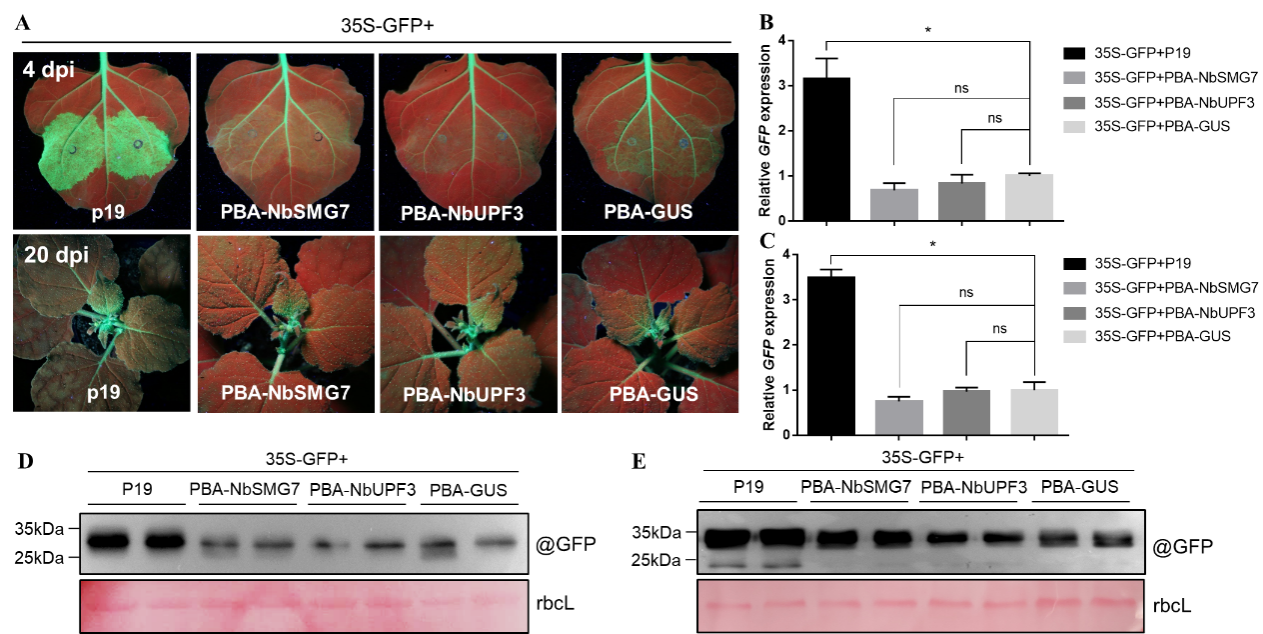


**Fig S1. Expression of NbSMG7 or NbUPF3 fails to suppress GFP-induced RNA silencing and degrade GFP RNA in *N. benthamiana* plants.** (**A**) Pictures of representative agroinfiltrated leaves or plants were taken at 5 dpi (upper panel) and 20 dpi (lower panel) under UV light. Leaf patches of 16c transgene *N. benthamiana* plants were agroinfiltrated with two vectors, including 35S-GFP, and one of the following vectors: Myc-tagged-GUS, Myc-tagged-NbSMG7 or NbUPF3, and TBSV p19 (B). Similar results were obtained from three independent experiments. (**B, C)** Analyses of relative accumulations of *GFP* mRNAs by specific qRT-PCR in the infiltrated leaves are shown in (A) at 5 dpi and 20 dpi. *NbActin* serves as an internal standard. Each mean value was calculated based on three independent experiments (n=3 samples). Values represent the mean ± SD. The asterisk indicates a significant difference compared to 35S-GFP+p19 (**P* < 0.01, Student's *t*-test). (**D, E**) Accumulations of GFP protein, Myc-tagged-NbSMG7, or NbUPF3 protein. Protein levels were analyzed by immunoblot analysis using antibodies against GFP. The RbcL was stained with Ponceau S to indicate equal protein loading.


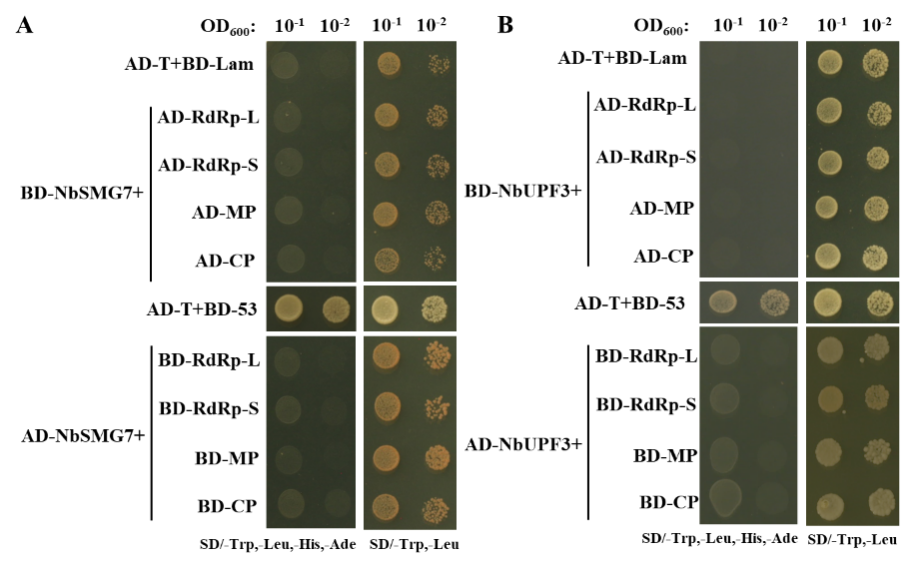


**Fig S2.** (**A, B**) Y2H Gold yeast strains co-transformed with the indicated plasmids were subjected to 10-fold serial dilutions and plated on SD/-Trp, -Leu, -His, -Ade medium to identify protein interactions at 3 days after transformation. Cells co-transformed with AD-T+BD-53 or AD-T+BD-Lam are positive or negative controls, respectively.


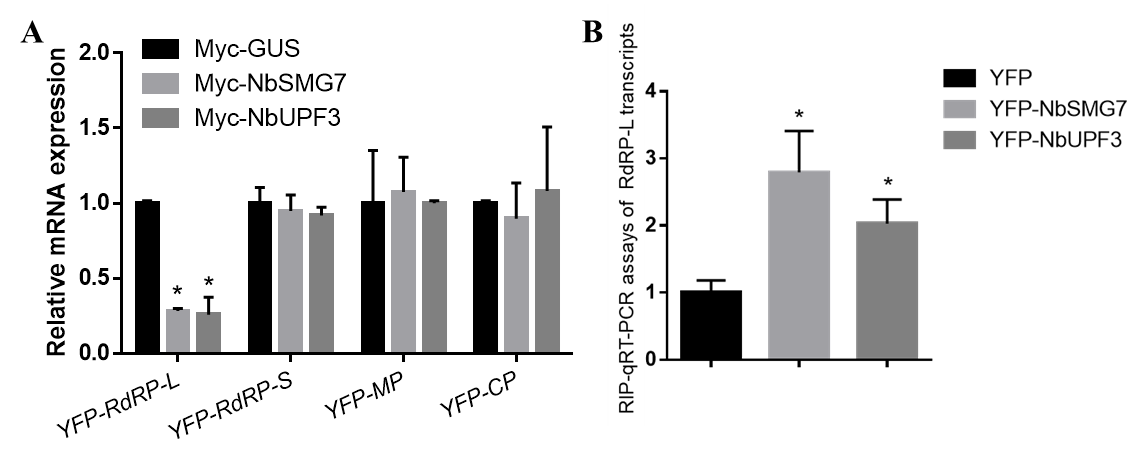


**Fig. S3** (**A**) qRT-PCR analysis of RNA accumulation of YFP-RdRP-L, YFP-RdRP-S, YFP-MP, and YFP-CP during expression of Myc-GUS, NbSMG7 and NbUPF3 at 60 hpi. (**B**) RIP-qRT-PCR assays of NbSMG7 and NbUPF3 associating with RdRP-L. The error bars indicate mean ± SD (n = 3); asterisks indicate a statistically significant difference according to Student's t-test (**P < 0.01; *P < 0.05), and ns means no significant difference.


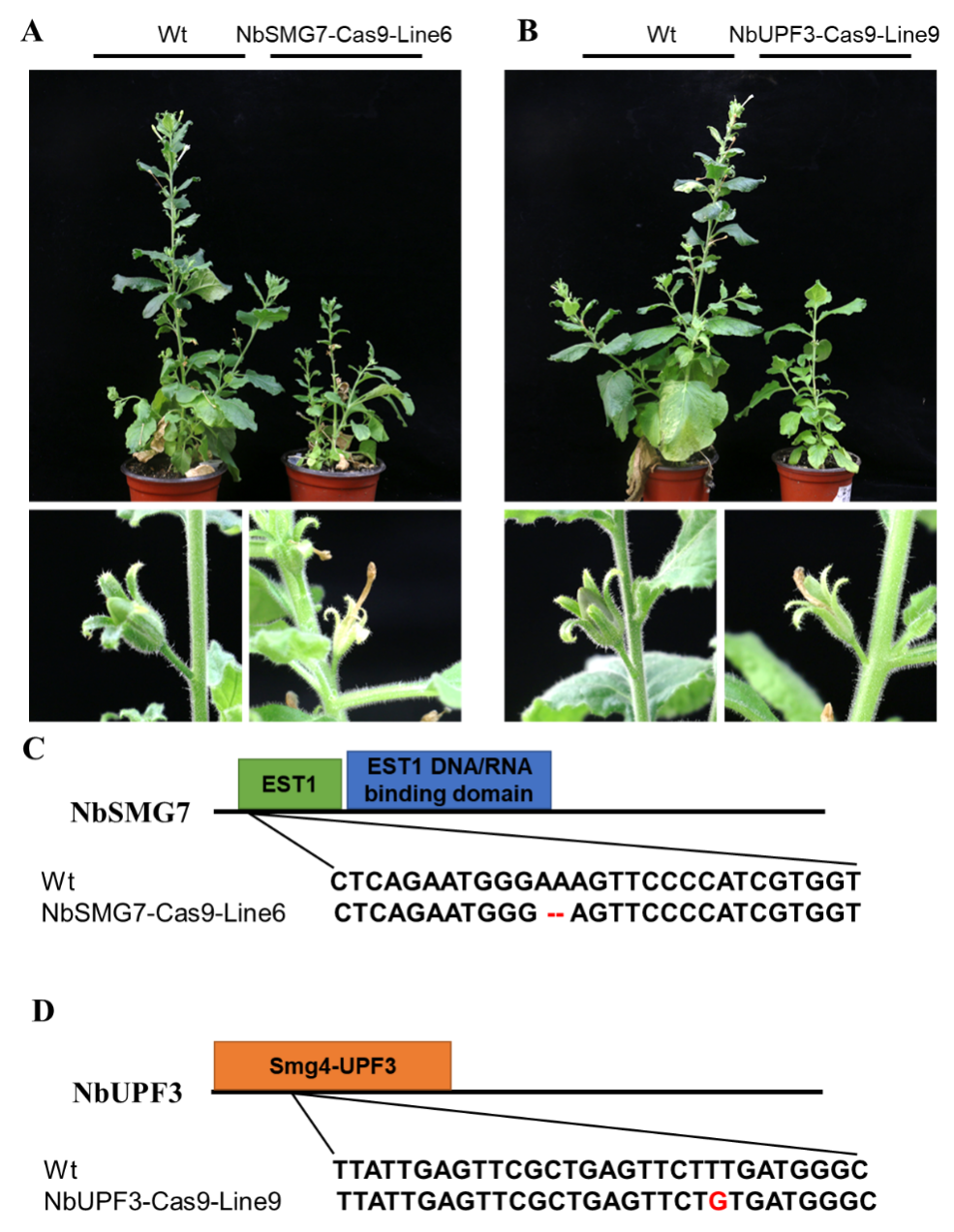


**Fig S4. Transgenic *N. benthamiana* plants with the knockout of *NbSMG7* or *NbUPF3*. (A-B)** The phenotype of transgenic NbSMG7-Cas9-Line6 (A), NbUPF3-Cas9-Line9 (B), and wild type (Wt) *N. benthamiana*. Knockout of *NbSMG7* or *NbUPF3* shows developmental defects, including dwarfing and sterile. (**C-D)** Sequence analysis of plants in (A-B) by PCR amplicons of gDNA of NbSMG7-Cas9-Line6 (A) and NbUPF3-Cas9-Line9 (B). Schematic representation of genome mutation of *NbSMG7* or *NbUPF3* locus in transgenic plants. Nucleotide mutation and deletion were found by sequencing analysis.


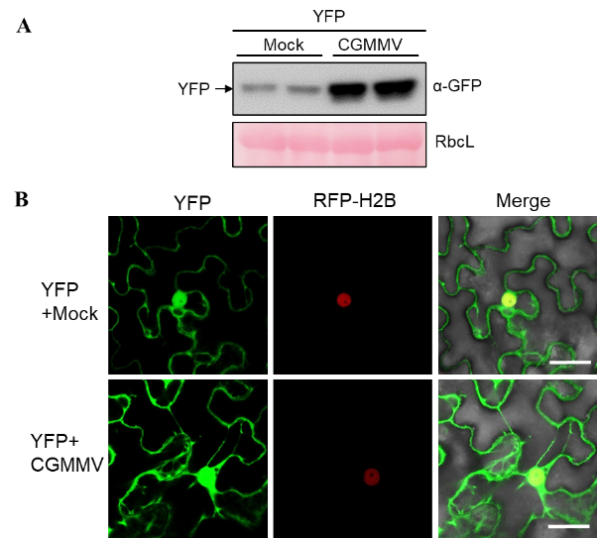


**Fig S5. The infection of CGMMV increases YFP accumulation.** RFP-H2B transgene *N. benthamiana* leaves were infiltrated with *Agrobacterium* cultures to express YFP alone or together with CGMMV. (**A**) Total proteins were detected with anti-GFP antibodies. Immunoblotting assays in this figure were repeated at least three times, and each experimental sample was derived from three individual infiltrations. One representative immunoblotting is shown. The RbcL was stained with Ponceau S to indicate equal protein loading. (**B**) The infiltrated RFP-H2B transgene *N. benthamiana* leaves were examined by confocal microscopy at 48 hpi. Three independent infiltrations repeated these experiments three times, and more than 20 cells per sample were observed in each replicate; representative results are shown. Scale bar: 50 µm.


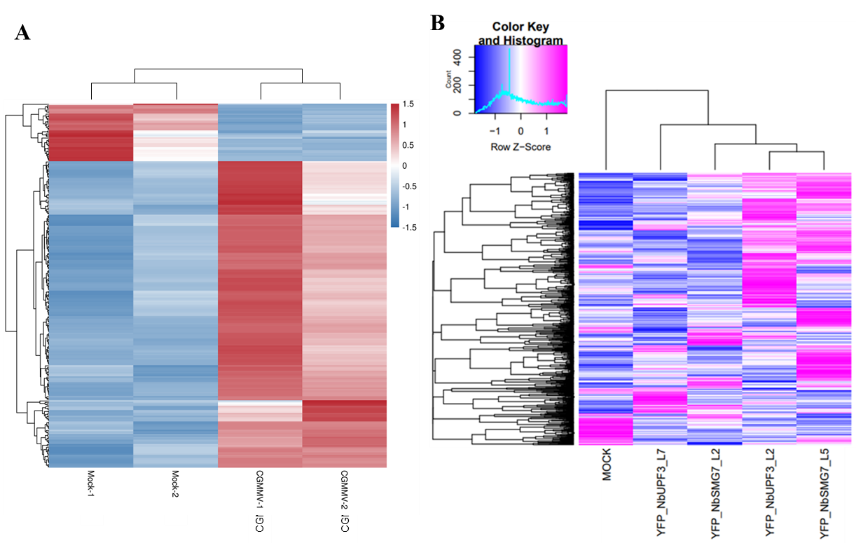


**Fig. S6 Heatmap representing the differentially expressed genes involved in transcription factors.** (**A-B**) Mock and CGMMV-infected *N. benthamiana* plants at 12 dpi, and In Wt, YFP-NbSMG7, and YFP-NbUPF3 transgenic *N. benthamiana* plants after seeding 40 dpi.


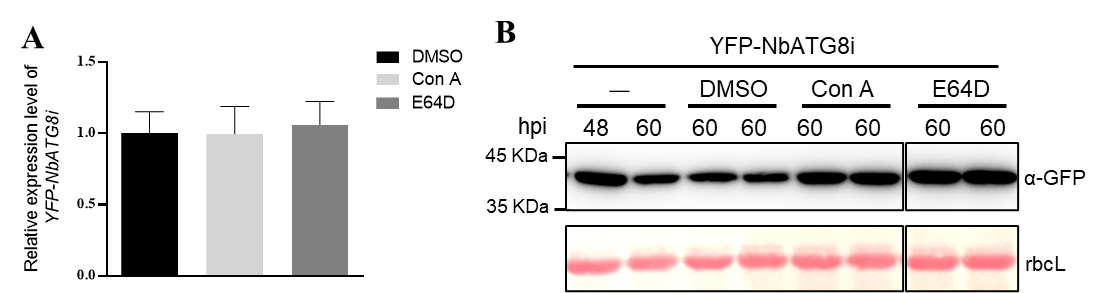


**Fig. S7 The effect of the autophagy inhibitor Con A, or E64D on the accumulation of YFP-NbATG8i RNA and protein.** (**A**) qRT-PCR analysis of the transcription level of *YFP-NbATG8i.* YFP-NbATG8i expressing leaves at 48 hpi were further treated with DMSO, Con A, or E64d, and then collected at 12 h after treatment as to 60 hpi for RNA extraction and RT-qPCR analysis. NbActin served as an internal standard. (**B**) Infiltrated leaves with YFP-NbATG8i were harvested at 48 hpi and 60 hpi, and YFP-NbATG8i expressing leaves at 48 hpi were further treated with DMSO, Con A, or E64d, and then collected at 12 h after treatment as to 60 hpi. Total protein was extracted from these samples. Western blot was conducted using an anti-GFP antibody. Ponceau S staining of Rubisco large subunit served as a loading control for western blot.


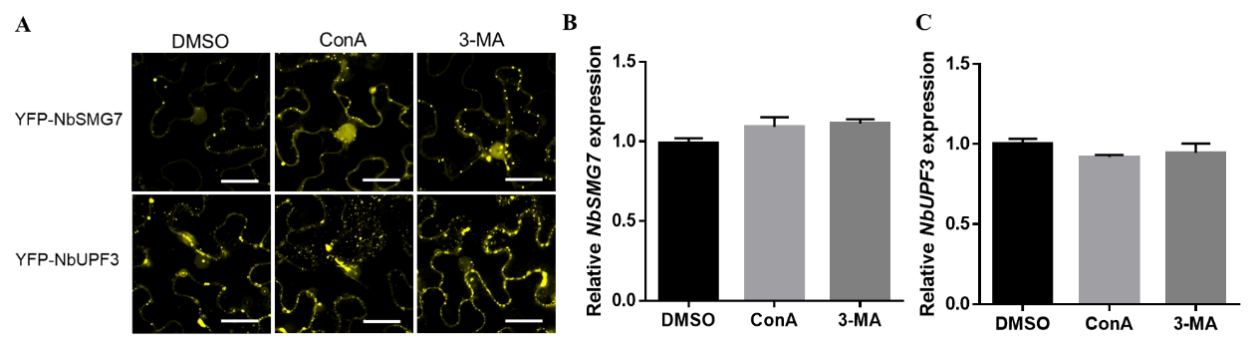


**Fig. S8 The effect of the autophagy inhibitors 3-MA and Con A on the accumulation of YFP-NbSMG7 and YFP-NbUPF3.** (**A**) CGMMV with YFP-NbSMG7 or YFP-NbUPF3 co-inﬁltrated leaves were treated with DMSO or 3-methyladenine (3-MA) or concanamycin A (Con A) after 36 hpi and confocal images were taken at 6 h after treatment. Bars, 25 μm. (**B**-**C**) qRT-PCR analysis of the expression of YFP-NbSMG7 (B) and YFP-NbUPF3 (C) in the (A)-infiltrated leaves at 42 hpi. The values of YFP-NbSMG7 (B) and YFP-NbUPF3 (C) were normalized against *NbActin* transcripts in the same sample. Three independent experiments, each consisting of three biological replicates, were carried out. Values from one representative result were used to plot a histogram. Statistical analysis (ns means no significant difference) was performed using Student's *t*-test.


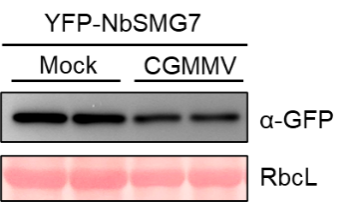


**Fig. S9 CGMMV decreased the protein accumulation of YFP-NbSMG7 in YFP- NbSMG7 transgenic *N. benthamiana*. Immunoblotting was performed using anti-GFP antibodies.**


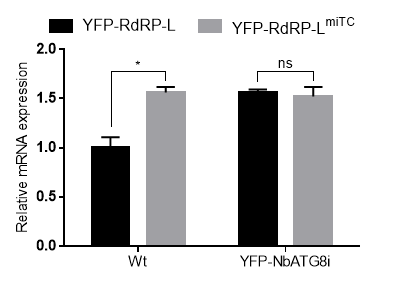


**Fig. S10 qRT-PCR analysis of RdRP-L and RdRP-L^miTC^ RNA accumulation levels in wild-type (Wt) and YFP-NbATG8i transgene *N. benthamiana* plants at 48 hpi.**

Supplementary Table1

| Primers | Sequence |
| --- | --- |
| 221/CGMMV/RdRPL/F | GGGGACAAGTTTGTACAAAAAAGCAGGCTTCATGGCAAACATTAATGAA |
| 221/CGMMV/RdRPL/R | GGGGACCACTTTGTACAAGAAAGCTGGGTCCTTAGAGACATCTATGTAAAGAC |
| 221/CGMMV/RdRPS/F | GGGGACAAGTTTGTACAAAAAAGCAGGCTTCATGGCAAACATTAATGAA |
| 221/CGMMV/RdRPS/R | GGGGACCACTTTGTACAAGAAAGCTGGGTCTTTGGTAGGCACAGTGGTA |
| 221/CGMMV/MP/F | GGGGACAAGTTTGTACAAAAAAGCAGGCTTCATGGCAAACATTAATGAACAAATC |
| 221/CGMMV/MP/R | GGGGACCACTTTGTACAAGAAAGCTGGGTCGGTGTGATCGGATTGTAA |
| 221/CGMMV/CP/F | GGGGACAAGTTTGTACAAAAAAGCAGGCTTCATGGCTTACAATCCGATCAC |
| 221/CGMMV/CP/R | GGGGACCACTTTGTACAAGAAAGCTGGGTCAGCTTTCGAGGTGGTAGCCT |
| 221/NbSMG7/F | GGGGACAAGTTTGTACAAAAAAGCAGGCTTCATGATGACCATTCCAATGGATAG |
| 221/NbSMG7/R | GGGGACCACTTTGTACAAGAAAGCTGGGTC CACAAACAAACGGCCTTC |
| CGMMV/qPCR/F | TACCCTCGTCTGTCGTAGATATT |
| CGMMV/qPCR/R | TGAGAAGCGAAACGAAGATAGG |
| TRV2/NbSMG7/BamHI/F | CGGGATCCAGCAAGTTCAGCGCCTCTACA |
| TRV2/NbSMG7/XhoI/R | CCGCTCGAGTGACGCGATCAGGTCCACC |
| NbSMG7/qPCR/F | GGCTTTGGTTCTGTTCCTTTG |
| NbSMG7/qPCR/R | CATCCAGCCAGCTATAGTCATC |
| NbSMG7/mAIM1/F | AGTGGGGCCAAGTTTGCCGATGCTGACAAGGATGATACGTGC |
| NbSMG7/mAIM1/R | GGCAAACTTGGCCCCACTAGACAAAAGCTTATTAAAGAAAGC |
| NbSMG7/mAIM2/F | AGACCTGCCAGGCATGCCGGCCCGCCTCCAGGCTTTGGT |
| NbSMG7/mAIM2/R | GGCATGCCTGGCAGGTCTGCTAACTGGATTTTTCCTTATGCC |
| 221/NbUPF3/F | GGGGACAAGTTTGTACAAAAAAGCAGGCTTCATGAAGAATCGGCGAACC |
| 221/NbUPF3/R | GGGGACCACTTTGTACAAGAAAGCTGGGTCAGAACCAGAACTTGACTTTTGA |
| TRV2/NbUPF3/BamHI/F | CGGGATCCATTAATGGATTATATACGTCAGA |
| TRV2/NbUPF3/XhoI/R | CCGCTCGAGTCCCCTTCCACTACATCA |
| TRV2/NbUPF3/EcoRI/F | CGGAATTC ATTAATGGATTATATACGTCAGA |
| TRV2/NbUPF3/BamHI/R | CGGGATCC TCCCCTTCCACTACATCA |
| NbUPF3/qPCR/F | CACATCCTCAAGAACTCCTAGC |
| NbUPF3/qPCR/R | CCTTAGCATTTCCAGCCTTAGA |
| 221/NbATG8i/F | GGGGACAAGTTTGTACAAAAAAGCAGGCTTCATGGGGAAGGCTTTCAAAA |
| 221/NbATG8i/R | GGGGACCACTTTGTACAAGAAAGCTGGGTCTCAACTATTTGCACGACCAAAG |
| TRV2/NbATG8i/EcoRI/F | CGGAATTCCCGTTGGCCAATTTATCCA |
| TRV2/NbATG8i/BamHI/R | CGGGATCCTCAACTATTTGCACGACCAAAGG |
| NbATG8i/qPCR/F | TTCAGACGATGAGAGACTCGCAGAATC |
| NbATG8i/qPCR/R | TTCCCAGGAGCCAGATGGAGTC |
| 35S-F | CGCAAGACCCTTCCTCTATATAAGGAA |
| T7 | TAATACGACTCACTATAGGG |
| M13F | TGTAAAACGACGGCCAGT |
| M13R | CAGGAAACAGCTATGACC |
| NbActin/qPCR/F | AAAGACCAGCTCATCCGTGGAGAA |
| NbActin/qPCR/R | TGTGGTTTCATGAATGCCAGCAGC |
| CGMMV/RdRPL/miTC/F | ACCAAAATGCAATTAATGCAGAACTCACTGTATGTCC |
| CGMMV/RdRPL/miTC/R | TAATTGCATTTTGGTAGGCACAGTGGTAGCAAACATAGT |

**Table S1.** A list of primers used in this study.


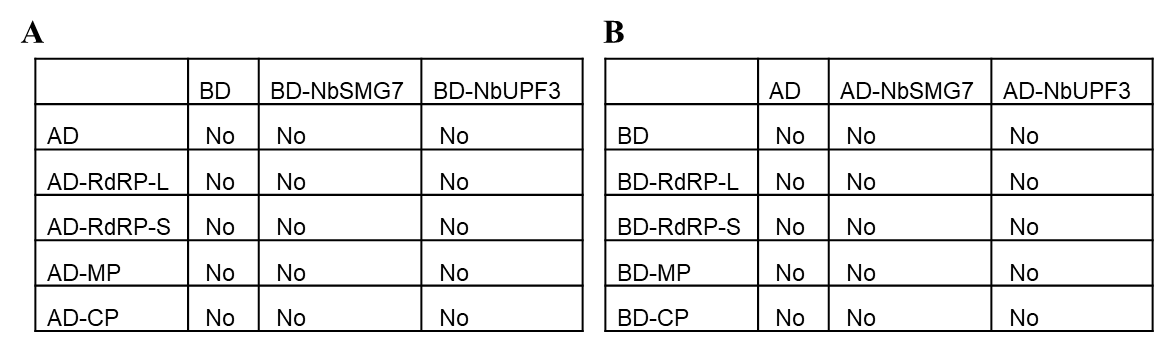


**Table S2. Summary of Y2H assay results**. Y2H Gold yeast strains co-transformed with the indicated plasmids were plated on SD/-Trp, -Leu, -His, -Ade medium to identify protein interactions 3 days after transformation. Proteins were fused to the Gal4 DNA binding (BD) or activation (AD) domain. No means no positive interaction between two tested proteins.


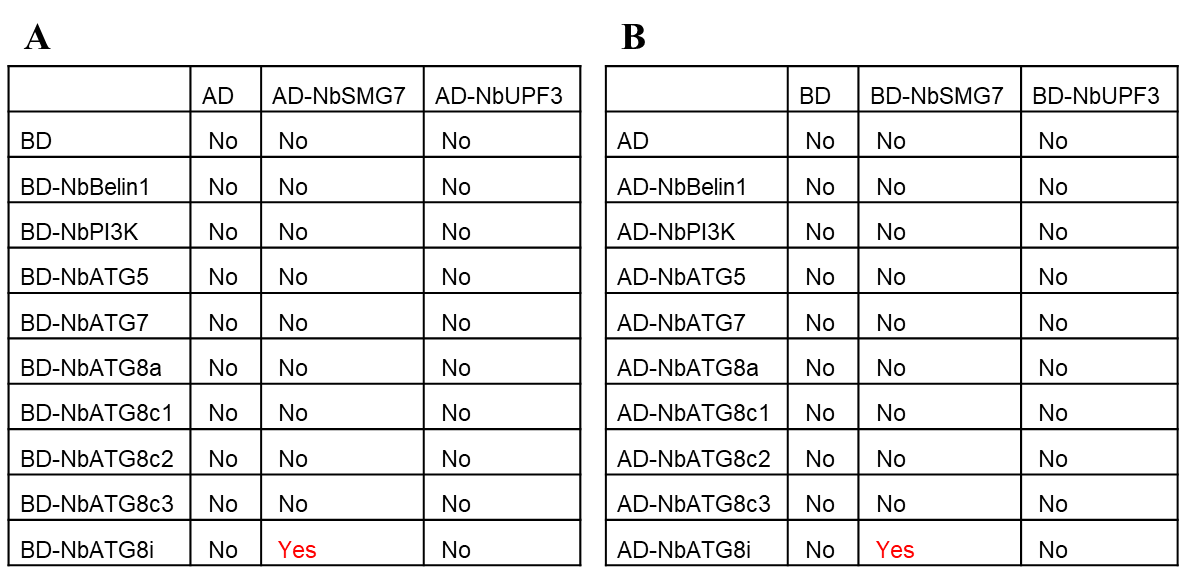


**Table S3.** **Yeast-two hybrid (Y2H) assays for possible interactions between NbSMG7 or NbUPF3 and nine autophagy-related proteins.** NbSMG7, NbUPF3, and nine ATGs-encoding proteins were fused with a GAL4 activation domain (AD) and a GAL4 binding domain (BD). Y2H Gold yeast cells co-transformed with the indicated plasmids were subjected to 10-fold serial dilutions and plated on synthetic dextrose (SD)/-Trp/-Leu/-His/-Ade medium to screen for positive interactions at 3 days after dilution. No means no positive interaction between two tested proteins, and Yes means positive interaction between two tested proteins.
